# A comparative computational analysis of IFN-alpha pharmacokinetics and its induced cellular response in mice and humans

**DOI:** 10.1371/journal.pcbi.1013509

**Published:** 2025-09-25

**Authors:** Priyata Kalra, Bastian Kister, Rebekka Fendt, Mario Köster, Julia Pulverer, Sven Sahle, Lars Kuepfer, Ursula Kummer

**Affiliations:** 1 Department of Modelling of Biological Processes, COS/BioQuant, Heidelberg University, Heidelberg, Germany; 2 Institute for Systems Medicine with Focus on Organ Interaction, University Hospital RWTH Aachen, Aachen, Germany; 3 Model System for Infection and Immunity, Helmholtz Centre for Infection Research, Braunschweig, Germany; University of California Riverside, UNITED STATES OF AMERICA

## Abstract

Drug effects are difficult to investigate in detail *in vivo*. However, a mechanistic understanding of drug action is clearly beneficial for both pharmaceutical development as well as for optimization of treatment designs. We here established a quantitative systems pharmacology (QSP) mouse model which simultaneously describes whole-body pharmacokinetics of murine IFN-α as well as the cellular pharmacodynamic effect through the antiviral response biomarker Mx2. To this end, a dynamic model of intracellular IFN-α signalling in the JAK/STAT pathway was combined with a whole-body physiologically-based pharmacokinetic model of IFN-α in mice. The pharmacodynamic behaviour of the resulting mouse IFN-α QSP model was first compared to a cellular model of the JAK/STAT pathway to compare *in vitro* and *in vivo* drug effects and to identify functional differences. It was found that the *in vitro* drug effect in the cellular model overestimates the *in vivo* response in mice at least by a factor of two which is partly due to the missing drug clearance *in vitro*. Also, the drug responses in the *in vitro* model were time delayed. Interspecies analyses in murine and a previously published human QSP model of IFN-α next show a similar dynamic behavior. However, our models demonstrate eight to 16-fold stronger response levels in mice than in humans due to more efficient interferon binding. Our analysis supports a mechanistic analysis of both upstream pharmacokinetic as well as downstream pharmacodynamic drug effects through the combination of physiological knowledge and quantitative computational models. The study hence shows potential applications for QSP modelling in terms of study planning, for example by choosing physiologically relevant in vitro concentrations. Also, the QSP model allows inter-species comparisons of the effect strength in specific functional readouts, which in humans are otherwise not possible due to the limited sampling possibilities. We expect QSP modelling to play an increasingly important role in drug development and research in the future.

## Author summary

We here established a computational mouse model which simultaneously describes whole-body pharmacokinetics of interferon alpha (IFN-α) after injection as well as the subsequent cellular response to the drug in liver cells, i.e., the resulting pharmacodynamic effect.

The simulated pharmacodynamic behaviour was first compared to an i*n vitro* cellular model (modeling the cellular response in cell culture) of the JAK/STAT pathway to compare *in vitro* and *in vivo* drug effects and to identify functional differences. It was found that the *in vitro* drug effect in the cellular model is stronger per unit administered IFN than the *in vivo* response in mice which is partly due to the missing drug clearance *in vitro*. In a second analysis, interspecies analyses of the new mouse model with a previously published corresponding human model show stronger response levels in mice than in humans per unit drug due to a more efficient interferon binding in mouse hepatocytes predicted by the model. Our analysis supports a mechanistic analysis of both upstream pharmacokinetic as well as downstream pharmacodynamic drug effects through the combination of physiological knowledge and quantitative computational models.

## Introduction

Therapeutic drug effects are governed by both drug disposition at the whole-body level and the resulting drug action at the cellular scale. Drug disposition is determined through drug ADME (ADME: absorption, distribution, metabolism and excretion) and the resulting drug pharmacokinetics (PK) [1]. Drug action itself reflects the molecular on-target interactions which ultimately lead to an observed pharmacodynamic (PD) response [2]. PK profiles can be measured in systemic blood through direct sampling. However, time-concentration data in specific tissues are largely restricted to animals due to invasive sampling and corresponding ethical concerns. Drug plasma concentrations can be used to approximate average target exposure. However, potential non-linear effects such as target-mediated drug disposition may require more elaborated tools such as physiologically-based pharmacokinetic (PBPK) modelling to quantify for example on-target drug disposition [3,4]. PD responses can be quantitatively analyzed at a large level of detail with specific *in vitro* assays. Still, the translation of these results to an *in vivo* situation may be erroneous due to a limited translatability of *in vitro* to *in vivo* conditions. Similar challenges hold for preclinical animal models which are often not comparable to human physiology. Here, it is also desirable to establish adequate PK/PD models that allow an early comparison of animal models with respect to human physiology and thus could support early decision-making if an animal model is adequate or which differences are to be expected. A truly comprehensive PK/PD understanding of a compound under *in vivo* conditions is hence difficult to achieve and inevitably inhibits several weaknesses which are also reflected by the continuously high attrition rates in pharmaceutical development [5].

Computational modelling carries the promise to overcome at least some of these limitations by integrating mechanistic knowledge in a mathematical framework. Such computational models ideally integrate both drug PK and drug PD in a structural representation. PK/PD models are also referred to as QSP models (QSP: quantitative systems pharmacology), in particular if the drug effect is considered at network level and not for a single isolated target [6]. QSP models provide the unique possibility to mechanistically correlate drug doses with a to-be-expected drug response. They may also be used to simulate cellular models based on *in vitro* data within an *in vivo* context at the whole-body level. Thus, QSP models may also simulate clinical response markers [7,8] to predict clinical efficacy. Also, human and animal QSP models can be systematically compared to identify inter-species differences in PK/PD behaviour. They are therefore important tools for pharmaceutical development in terms of study design or decision-making.

**Fig 1:**
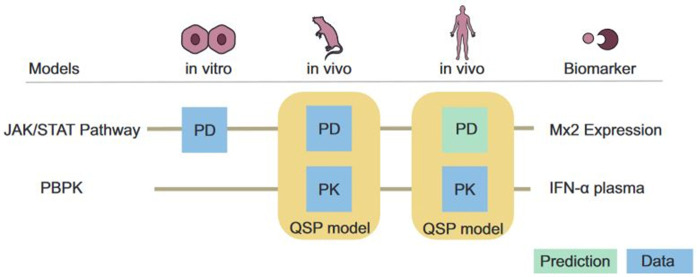
QSP models of intravenous IFN-α administration in mice and men. Provision of a novel quantitative cross-species extrapolation approach based on a mechanistically detailed QSP models of intracellular responses via JAK/STAT pathway in human and mice.

**Fig 2:**
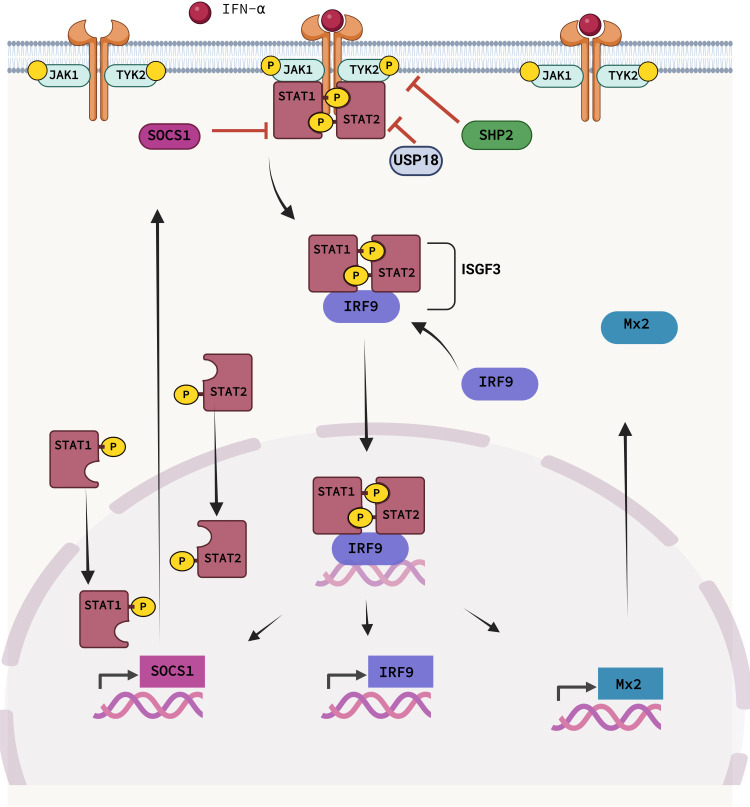
Illustration of the canonical JAK/STAT pathway. The key steps of the pathway in response to IFN-α are highlighted. IFN binds to the receptor and activates JAK1 and TYK2, which subsequently activate the STATs in the cytoplasm, all by phosphorylation. The phosphorylated STATs and IRF9 form the transcription complex ISGF3, which translocates to the nucleus, where it activates hundreds of ISGs, including IRF9, Mx2 and SOCS1. Heatmap summarizing all analyzed mouse-human correlations. E. Phase I metabolizing enzymes; and F. carriers differentially expressed in human liver disease.

To illustrate such translational concepts and to test and improve cross-species extrapolations, we here present a new QSP-based analysis of IFN-α treatment in mice and humans (Fig 1). The cytokine IFN-α is used for the treatment of various diseases, e.g., hepatitis C virus (HCV) infections and multiple sclerosis. It triggers the innate immune responses and helps liver cells clear viruses. We previously established a QSP model for humans [9] which we now transferred to mice. The model integrates a detailed cellular model of IFN-α signalling through the JAK/STAT pathway [10] (Fig 2) into a physiologically-based pharmacokinetic (PBPK) model at the whole-body scale.

Thus, it was possible to simultaneously describe IFN-α PK as well as the resulting drug-induced PD response within one integrated multiscale model representation. The integration of cellular models within whole-body PBPK models [3] provides the unique opportunity to simulate cellular responses to IFN-α stimuli within an *in vivo* context (Fig 1).

The resulting model for mice was used to compare *in vitro* and *in vivo* drug responses and for cross-species analysis using human model as a reference. According to the analysis, the hepatocellular anti-viral response would be expected to be stronger than the one in human hepatocytes when administering identical weight-normalized doses. Thus, using a mouse model to test the anti-viral response to IFN-α administration would overestimate the expected response in humans.

We expect multiscale QSP models as the one investigated in this study for IFN-α to become of increasing importance in pharmaceutical development in the future.

## Materials and methods

### Mice and ethics statement

In an analogue setting, transgenic mice were injected with a single i.v. dose of 2000 U mu-IFN-α-4 and Mx2 expression was measured as an average radiance from the whole liver at 3, 6, 9, 12, 24, 48 h post injection. Animals were handled in strict accordance with good animal practice as defined by the relevant local animal welfare bodies, and all animal work was approved by the appropriate committee (Niedersächsisches Landesamt für Verbraucherschutz und Lebensmittelsicherheit [LAVES], Oldenburg, Germany). All mice were bred under standard conditions at the Helmholtz Centre for Infection Research (Braunschweig, Germany).

### *Mx2-Luciferase mouse hepatocyte assay and in vivo* bioluminescence imaging

Murine hepatocytes were isolated from Mx2Luc transgenic mouse strain [11] by using a two step perfusion protocol [12,13]. Mice were narcotized with ketamine/xylazine (0.1 ml per 10 g body weight). The catheter was fixed in the portal vein and connected to the external pump system. Liver perfusion was processed by using 125 ml prewarmed (37°C) liver perfusion medium (Gibco) supplemented with heparin (2500 U/ml) with a flow rate of 8 ml/min. The vena cava inferior was opened to enable the emission of the medium. Enzymatic digestion was performed by application of 125 ml liver digest medium (Gibco) supplemented with 5 g Liberase (Roche), corresponding to 40 mg/ml with a flow rate of 25 ml/min. Upon complete digestion of the liver, the liver was resected and placed in a petri dish filled with precooled (4°C) Dulbecco’s Eagle Medium (DMEM) (Gibco) supplemented with 10% fetal bovine serum (FBS) (Lonza), 100 U penicillin (Gibco), 100 µg/ml streptomycin (Gibco), and 2 mM glutamine (Gibco). The liver lobes were carved and the hepatocytes were gently shaken out of the liver. The suspension was passed through a 100 µm cell strainer to remove liver tissue. Hepatocytes were purified and washed by centrifugation at 50 g, 4°C, for 5 min without break. The supernatant (non-parenchymal fraction) was discarded and the hepatocyte pellet was resuspended in the appropriate medium for cultivation. Primary hepatocytes were cultivated in cell culture dishes that were coated with 0.2% collagen solution (InSCREENeX GmbH) and were maintained at 37°C in a humidified atmosphere with 5% CO2. For cultivation of murine hepatocytes Dulbecco’s Eagle Medium (DMEM) (Gibco) supplemented with 10% FBS (Lonza), 100 U penicillin (Gibco), 100 µg/ml streptomycin (Gibco), and 2 mM glutamine (Gibco) was used. IFN-α4 was purchased from PBL Interferonsource.

For quantification of the enzymatic activity of luciferase, cells were lysed in passive lysis buffer (Promega). Cell extracts were assayed for luciferase activity using standard reaction buffer (20 mM glycylglycine, 12 mM MgSO4, 1 mM ATP) containing luciferin (SynChem OHG) and a single-tube luminometer (Berthold).

For *in vivo* bioluminescence imaging, mice were injected intraperitoneally with 3 mg of d-luciferin (Synchem OHG) in PBS and anesthetized using 2 to 2.5% isoflurane (Abbott). For IVIS we followed the instructions from the manufacturer of the anesthesia system to anesthetize mice. External oxygen supply was adjusted to 3.5 bar. Oxygen was delivered to the vaporizers at a constant pressure of 6 PSI. Anesthesia was induced and maintained using isoflurane (2-2.5%) in air, and animals were recovered afterwards with no ill effect. Flow rates were adjusted to 1 liters per minute. Animals were anesthetized and luciferin was subsequently injected intraperitoneal. Animals were transferred to the imaging platform and placed on a heating pad to maintain body temperature. 5 minutes after luciferin injection, imaging was performed for an additional period of 2–3 minutes. Following imaging, animals were monitored until fully recovered from anesthesia (walking, active, etc.). Bioluminescence was analyzed using the Caliper *in vivo* imaging system (IVIS-200). Data analysis was performed with Living Image 3.1 software (Caliper LifeSciences). Signal emitted by regions of interest was measured, and data were expressed as photon flux, quantified as photons s^1^ cm^2^ sr^1^. Residual IFN-α in the serum of i.v. injected mice. 5000 units of IFN-α4 were injected into C57BL/6 mice via tail vein. Blood was taken at the indicated time points and IFN-α in the serum was determined by EILSA (PBL Interferonsource).

The luciferase activity was quantified by measuring the emitted light intensity of the luciferin-luciferase reaction. In order to link the Mx2-Luciferase activity data to the Mx2 expression in the liver, a scaling parameter $\rho_{Mx\text{2}}$ was introduced in the model:


$${Mx\text{2}}^{L}(t)=\rho_{Mx\text{2}}*Mx\text{2}(t)$$ (1)

with ${Mx\text{2}}^{L}(t)$ denoting the Mx2-Luciferase activity and Mx2(t) the Mx2 expression in the liver. The scaling parameter was then fitted to the data set.

### Cell cultures

Primary mouse hepatocytes (PMH) isolated from transgenic mice were treated with different doses of mu-IFN-α-4. The reporter construct was expressed under the Mx2 promoter. Dose response expression was measured for 1, 10, 100, 500, and 2500 U/ml mu-IFN-α-4 at 24 hours. Time resolved measurements were then performed for 5 U/ml and 500 U/ml at time points 0, 4, 8, 12, 19, 23, 27, and 34.5 h. The specific activity of mu-IFN-α-4 was 1.4*10^8^ U/mg.

### Software

#### Copasi.

The cellular JAK/STAT signalling model of IFN-α was developed in Copasi [14], version 4.22, an open source software freely available at www.copasi.org.

#### PK-Sim.

The PBPK model of IFN-α was built using the PBPK modelling software PK-Sim® (Open Systems Pharmacology suite Version 7.2 available at www.github.com).

#### MoBi.

The PBPK protein model was established in PK-Sim® (Open Systems Pharmacology suite Version 7.0).

### PBPK modelling

In PBPK models, the physiological mechanisms governing the distribution of molecules within the body of an organism are explicitly represented [3]. PBPK models are based on compound properties such as molecular weight or lipophlicity as well as properties describing the physiology of the species considered. The compound properties can usually be measured with *in vitro* assays and are required to inform the distribution model to estimate tissue-plasma partition coefficients as well as permeabilties. Physiological parameters such as organ volumes or blood flows are usually taken from public databases or data repositories. They already are available in the PBPK software PK-Sim for species such as mice, rats, rabbits, dogs, monkeys or humans. Of note, PBPK modelling thereby supports cross-species extrapolation as well as interspecies comparisons.

### PD model of the JAK/STAT pathway

The development and structure of the JAK/STAT signaling pathway model largely follows earlier work [9] and is described in detail in S1 Text. A list of model reactions as well as model parameters is also provided in the supplementary material (Tables A and B in S1 Text).

The integration of the PBPK model with the cellular JAK/STAT signaling model was implemented in MoBi. Most reactions of the PBPK/PD model were described as mass action kinetics [15]. The basal receptor turnover of IFNAR1 and IFNAR2 were modelled as:


$$\text{v}={\text{rel}}_{\text{norm}\_\text{exp}}[{\text{S}}_{\text{ref}}{]}^{*}{\text{K}}_{\text{turnover}}-[\text{Substrate}{]}^{*}{\text{K}}_{\text{turnover}}$$ (2)

with rel_norm_exp_ denoting the normalized relative expression of the respective receptor in different organs and S_ref_ representing the reference concentration of the receptor. The feedback inhibition of SOCS1 on the receptors was modelled as irreversible catalytic activation on the receptor


$$\text{v}=\frac{V_{max}*[Substrate]*[Inhibitor]}{\left(k_{mx}+[Substrate]\right)\cdot \left(K_{a}+[Inhibitor]\right)}$$ (3)

Simulation of the models were performed using the LSODA algorithm as implemented in Copasi, as well as cvode 42 in PK Sim/MoBi. Parameter fitting was performed with the Particle Swarm and Hooke/Jeeves algorithms as implemented in Copasi for fitting intracellular dose-response behaviour and Monte Carlo/Levenberg-Marquardt implemented in the Open Systems Pharmacology suite (Matlab toolbox).

For comparing different models obtained by parameter estimation, the mean absolute error (MAE), the root mean squared deviation (RMSD) and the corrected Akaike information criterion AIC_c_ [16] was calculated as


$${AIC}_{c}(\chi^{\text{2}},n,k)=\text{2}k+\;\chi^{\text{2}}+\text{2}k\frac{k+\text{1}}{n-k-\text{1}}$$ (4)

by using the number of parameters k, the number of data points n and the achieved minimum of the cost function χ^2^. Models with the lowest index yield more information; however, those with similar indices are considered to be equally informative.

### PK-Data

Previously published PK data for administration of IFN-α and IFN-β were used for PBPK model development [17–19] (Table A in S2 Text). The time-concentration profiles were digitized by using WebPlotDigitizer (www.automeris.io) (Table B in S2 Text). Following best practice recommendations [3] for PBPK model building and validation, an initial model version was developed for two similar doses of IFN-α (4.43 µg [17] and 3.50 µg [19]) before two smaller doses (3.33 µg IFN-α [19]) and 0.0357 µg IFN-α/IFN-β (own data)) were simulated and compared to the corresponding experimental data. As an initial quality assessment, dose normalization was performed for all four datasets to ensure their comparability (Fig A in S2 Text).

### In vivo-in vitro comparison

To compare in vivo and in vitro responses, the administered in vivo dose of 1.43 µg IFN-α (corresponding to 2000 U) was converted to an effective liver interstitial concentration of 1.39 nM using the PBPK model. This concentration reflects the actual target site exposure accounting for systemic distribution, clearance, and tissue-specific kinetics. The intracellular model was then stimulated with this concentration to simulate the in vitro response.

## Results

In preclinical research, animal models are used to investigate the PK/PD behavior of a drug. As such, animal models are used to bridge between early *in vitro* screening and later clinical trials in human patients. However, this translational step has inherent uncertainties regarding the comparability of *in vitro* assays and animal models on the one hand and human patients on the other. To systematically address this question, we developed here a QSP model of IFN-α treatment in mice, which we validated by using Mx2 expression as an *in vivo* readout. We then compared the simulations of this validated mouse QSP model with further molecular markers of JAK/STAT signalling, as well as simulations of a previously published QSP model of IFN-α treatment in humans [9].

### Model set-up

We started our analysis with the development of a multiscale QSP model of IFN-α treatment in mice. In brief, model development included the following three steps:

1. Establishment of an intracellular PD model of the JAK/STAT signalling pathway in mice at cellular scale on the basis of a previously established human model [9],
2. Establishment of a PBPK model of interferon administration at the whole-body level in mice,
3. Integration of both models into a multiscale QSP model for mice.

In the following we describe the individual steps in more detail.

### Establishment of an intracellular PD model of the JAK/STAT signalling pathway in mice

The signalling cascade of IFN-α is initiated by the binding of IFN-α to the IFN receptor subunits IFNAR1 and IFNAR2 to form a heterodimeric ligand receptor complex. The receptor complex employs receptor bound phosphorylated Janus kinase 1 (JAK1) and Tyk2 to consequentially phosphorylate signal transductor and activator of transcription 1 and 2 (STAT1, STAT2) proteins. The phosphorylated STAT proteins (pSTAT1/pSTAT2) form a heterodimer which then further associates with interferon regulatory factor 9 (IRF9) to form transcriptionally active IFN-stimulated gene factor 3 (ISGF3). ISGF3 translocates into the nucleus and binds to interferon-stimulated response elements (ISREs). Activated ISREs modulate the expression of numerous interferon stimulated genes (ISGs), e.g., antiviral proteins like Myxovirus resistance (Mx1, Mx2) proteins and RNA-dependent protein kinase (PKR) [20]. Mx proteins, belonging to the GTPase superfamily, are highly conserved across species and are common markers for antiviral responses.

In humans, two variants of Mx proteins can be found, MxA and MxB, and the corresponding homologues in mice are Mx1 and Mx2 [21,22]. The JAK/STAT pathway is tightly regulated by many negative feedback regulators, of which, SOCS (suppressors of cytokine signalling), PIAS (protein inhibitors of activated stats) and PTPs (protein tyrosine phosphatases) are among the most important ones.

The model describing the pharmacodynamics of the JAK/STAT signalling pathway in mice was based on a previously established model for humans [9]. In particular, in that earlier work we had identified an ensemble of model parameterizations that all show equally good agreement of the model simulations with *in vitro* data [9]. To assess comparability between the human and murine pathway, we performed a BLASTP search for proteins of the JAK/STAT signalling pathway. Sequence identity and homology were found to be above 80% for most proteins of the signalling pathway, indicating a strong identity between proteins structures for the two species (Table C in S1 Text). We hence assumed most kinetic parameters, besides those for ligand-receptor binding (significant differences in protein sequence), and all initial concentrations to be identical to the human model. We furthermore adjusted the volume of the liver compartment to reflect the altered physiology in mice.

In order to address expression data of the biomarker Mx2 in response to IFN-α stimulus, we added three reactions describing transcription, translation and degradation of Mx2. A study by Asano et al. [23] showed that two ISRE binding sites and one IRF binding site are located within the Mx2 promoter region. We tested a model assuming cooperative binding of the transcription factors, but found that cooperativity was not important for describing Mx2 expression (Fig A in S1 Text). We therefore only used mass action kinetics to describe all reactions.

Parameters describing Mx2 expression were obtained by parameter estimation using all ten, previously published parametrizations of the human model [9] as a basis. Thus, these initial parameters were quite variable, since individual parameters had not been identifiable in the original human model and also contain parameters that simulate different expression levels of the individual proteins. Then, each of these parametrizations served as initial values for 300 new parametrizations fitting the mouse data and focusing on receptor kinetics and reactions of Mx2 expression, yielding a total of 3000 parameter sets.

To address the unidentifiability of most parameters of the intracellular signalling pathway, again, a model ensemble of the ten best fits were used for further analyses [24] (Table B in S1 Text). However, the final ensemble only differed with respect to their absolute Mx2 expression (Figs B and C in S1 Text). For simplicity, only simulations from one model are shown and discussed, but apply for the ensemble as a whole. Alternative, yet suboptimal ensembles are additionally provided in the supplementary material (Table D and Figs D and E in S1 Text).

Models were chosen such that they meet three criteria: (i) We selected only fits that performed best in the fitting procedure for time-resolved, dynamic data describing the Mx2-Luciferase activity; (ii) we verified that all models were able to reach a steady state without stimulation as well as with 5 U or 500 U IFN-α; (iii) models were evaluated on their ability to reproduce unseen dose response data for Mx2-luciferase activity at 24 h incubation time between 1 U and 2500 U of IFN-α.

**Fig 3:**
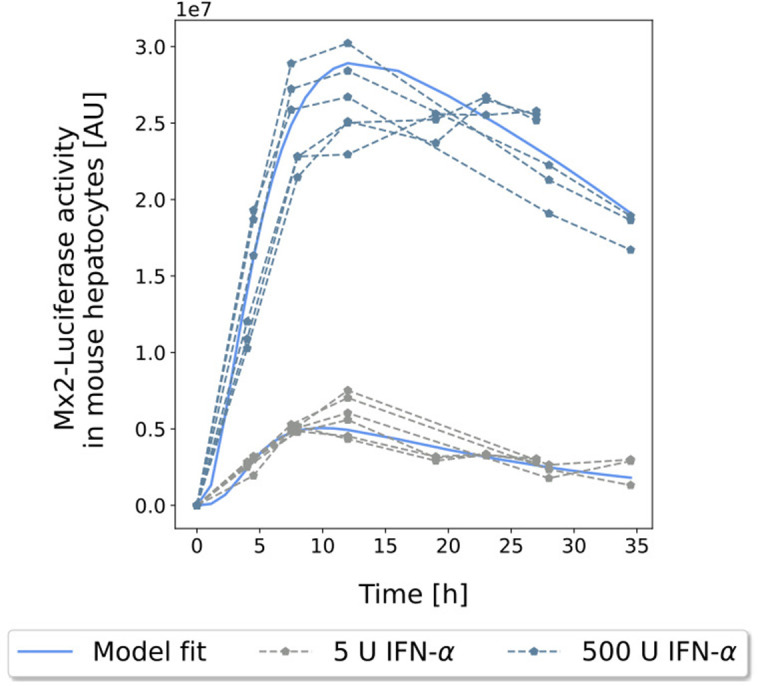
Fitted Mx2-expression data for the *in vitro* model ensemble. Predictions of the model for Mx2 expression in response to 5 U or 500 U IFN-α stimulation (solid lines) and corresponding data points used for fitting (dotted lines).

**Fig 4:**
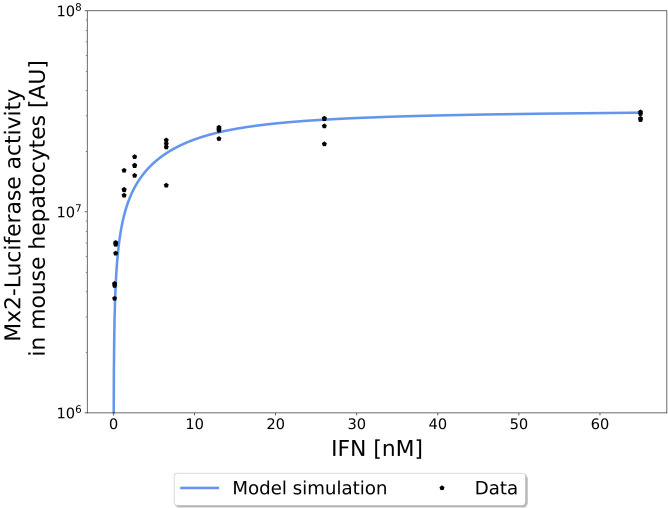
Predictions of the *in vitro* model ensemble for dose response data. Predicted dose responses of the model for Mx2 expression at 24 h after stimulation with 0.13 nM, 0.26 nM, 1.3 nM, 2.6 nM, 6.5 nM, 13 nM, 26 nM and 65 nM IFN-α. (corresponding to 5 U, 10 U, 50 U, 100 U, 250 U, 500 U, 1000 U and 2500 U IFN-α).

The final model captured the dynamics for Mx2-luciferase activity very well both for the high and the low dose, which differed by two orders of magnitude in the *in vitro* assay (Fig 3). The maximum concentration as well as its timing were slightly underestimated by all models. We also considered Mx2-luciferase activity after 24 h incubation time for various doses between 1 U and 2500 U of IFN-α. The computational simulations of the pathway responses for the different IFN-α doses are in excellent agreement with the experimentally measured Mx2-luciferase activity (Fig 4). This shows that the intracellular pathway model is capable to accurately describe an important endpoint of IFN-α signalling in the JAK/STAT pathway and can hence be used for further analyses.

### Establishment of a PBPK model of interferon administration in mice

**Fig 5:**
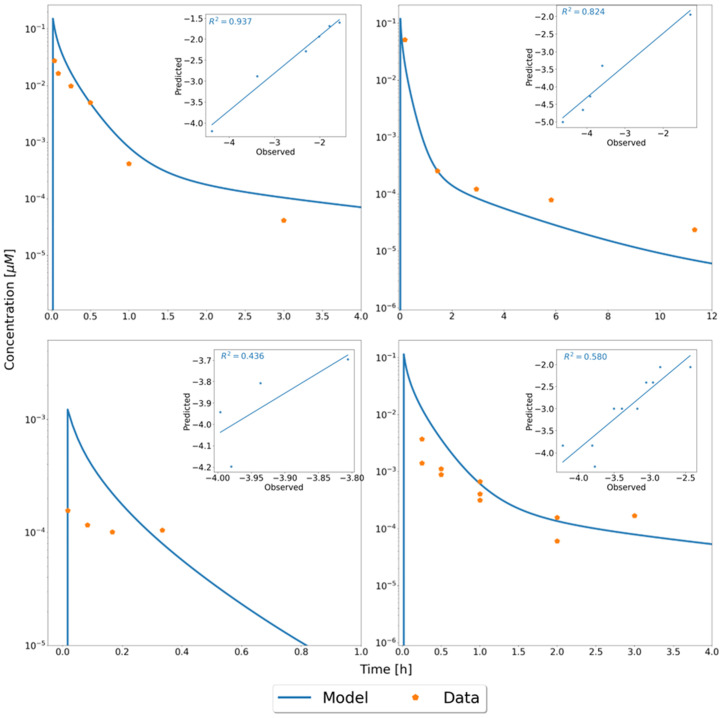
Development and validation of the mouse PBPK model to the venous blood plasma concentration. Model fits of the PBPK model to experimental data for IFN-α concentration in blood plasma after i.v. injection of a single dose of 4.43 µg or 3.5 µg IFN- α from Bohoslawec et al. (upper left, [17]) or Rosztoczy et al. (upper right, [19]), respectively, and model validation against Kiuchi et al. (lower left, mix of IFN- α/IFN-β and a data set produced for this study (lower right) after i.v. injection of a single dose of 3.33 µg or 0.0357 µg IFN- α respectively.

To describe drug disposition at the whole-body level, we developed a PBPK model of murine IFN-α in mice [3,25]. Previously published mouse PK data after IFN-α administration [17–19] as well as own data was used for model building and validation, following general practice guidelines for model development [3]. Dose normalisation of PK data was used for assessment of data consistency and showed an general comparability of all data (Fig A in S2 Text). The PBPK model was fitted to plasma concentrations after i.v. injections of a single doses of 4.43 µg and 3.50 µg IFN-α from Bohoslawec et al. [17] and Rosztoczy et al. [19], respectively (Fig 5). Plasma clearance, the individual receptor and receptor complex dissociation rates and dissociation constants K_off_ and K_D_, the endocytosis rate of the receptor complex into the cells and the basal turnover rates of the individual receptors were obtained by parameter estimation. Additionally, renal clearance was also considered in the mouse PBPK model. After the initial parameter estimation, both the data set from Bohoslawec et al. [17] and the one from Roszotoczy et al. [19] could be well described with the mouse PBPK model (Fig 5, R^2^ = 0.937 and R^2^ = 0.824, respectively).

Subsequently, model validation was performed with own data as well as from Kiuchi et al. [18] (Fig 5). In these studies, plasma concentrations of murine IFN-α were measured after i.v. injection of a single dose of 0.0357 µg IFN-α/IFN-β (own data) or 3.33 µg IFN-α [18], respectively. We found that the high dose of 3.33 µg IFN-α could be well described with the model (R^2^ = 0.580). However, the simulations for the lower dose, which was about a hundred fold lower than the dose used for model establishment, showed a large deviation from the experimental data (R^2^ = 0.436), in particular due to an unexpected plateau in the concentrations.

### Integration of both models in a multiscale QSP model of IFN-α drug action in mice

After developing and validating both the PBPK model and the PD model of IFN-α both models were combined to obtain a multiscale QSP model. The pharmacodynamic model was integrated into the interstitial space of the liver. The interface between the PBPK and PD model was the binding of IFN-α to its receptor at the target site at the extracellular target site. The compartmentalisation and structure of the PD model was unchanged in the multiscale model. Using the non-linear pharmacokinetic behaviour (absorption, distribution, metabolism and elimination) of IFN-α as an input, signalling behaviour could therefore be predicted in an *in vivo* context.

**Fig 6:**
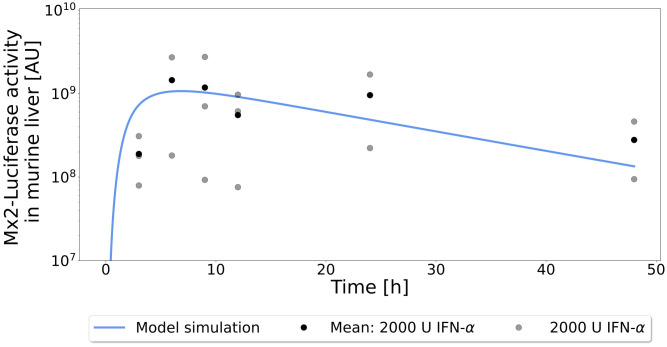
Model fit and validation for Mx2 activity. Predictions of the model for the expression of Mx2 in response to 1.43 µg murine IFN-α (solid lines) and data on Mx2-Luciferase activity in the liver of mice (points). Mean values are depicted as black points and were used for fitting. Individual measurements were displayed as gray points to show variation in the data.

For validation, the here established murine QSP model was tested against data of Mx2-Luciferase activity in mice. The predicted dynamics of Mx2 expression *in vivo* matched the observed data very well, especially considering that the data spanned up to two orders of magnitude (Fig 6). Although the initial time point at 3 h was slightly overestimated, the remaining time points could be reproduced very well.

Since not a single model parameter has been adjusted during model integration in the final QSP model, the correct description of the *in vivo* MX2-luciferase activity is noteworthy. In particular during the clearance phase after 12h the simulations are in excellent agreement with the experimental data. During this phase IFN-α pharmacokinetics and target-mediated clearance dominate the dynamics of the data.

### In vitro - in vivo comparison of IFN-α drug action

A key step in pharmaceutical research is the translation of *in vitro* results to the in vivo situation. However, the relevance of experimental findings from the laboratory to the situation in living organisms may be limited. The QSP model established here for mice allowed us to quantitatively compare MX2 expression according to a full mechanistic intracellular JAK/STAT model based on *in vitro* data with the drug-induced *in vivo* response in mice. In this regard, an obvious difference are the upstream IFN-α time concentration profiles, which are stationary in the *in vitro* case yet highly dynamic in *in vivo* situation due to the governing PK profile as governed by systemic clearance reactions.

For this, the QSP model was simulated with a single i.v. dose of 1.43 µg IFN-α as considered before in the intracellular model. This resulted in a maximum concentration of 1.39 nM in the liver. This was then used as the stimulus for the isolated PD model as representation of the *in vitro* cell culture experiment.

**Fig 7:**
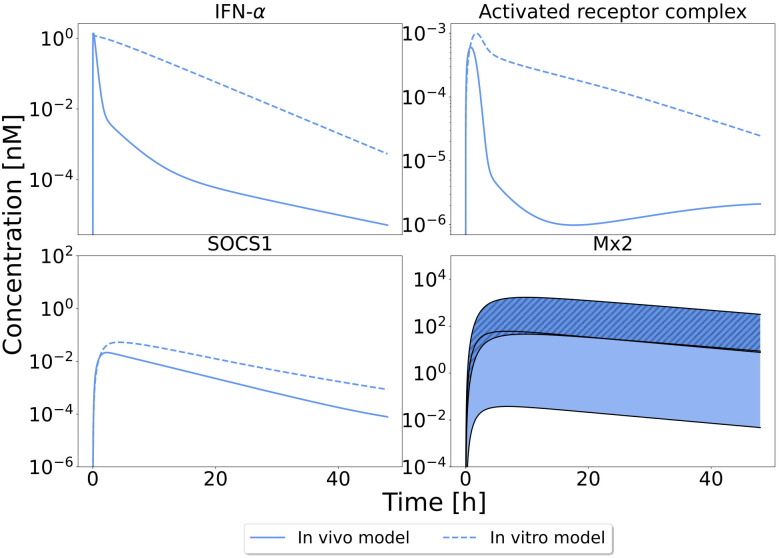
*In vitro* - *in vivo* difference in signalling patterns. Differences in signalling patterns in the *in vivo* QSP response (1.43 µg injected dose solid lines) vs the *in vitro* PD response (0.695 model units; initial dose; dashed lines). Displayed are the time profiles of IFN-α in the interstitial space of the liver, the activated receptor complex, the negative regulator SOCS1 and the target protein Mx2. For the first three, the ensemble predictions coincide; for Mx2 levels, the range of the ensemble’s predictions are shown.

The predictions showed in general more sustained levels of IFN-α at the target site in the *in vitro* setting than in the *in vivo* mouse model (16-fold increased AUC). As a consequence, also downstream signalling showed a stronger and prolonged activation profile compared to the *in vivo* model predictions. This means that the drug-induced response *in vitro* overestimates the actual *in vivo* behavior (Fig 7) and this can lead to erroneous conclusions regarding drug action as well as drug efficacy. Obviously, the computational model can be directly applied in clinical translation to overcome such misinterpretations.

To further evaluate *in vitro* - *in vivo* differences of IFN-α drug action, fold changes of predictions from the *in vitro* model to the *in vivo* context were calculated for various molecular species of the JAK/STAT signalling pathway (Fig A in S3 Text). This analysis showed, for all components of the cascade, a stronger activation in the *in vitro* setting for both maximal concentration (C_max_ two to eight-fold) and integrated activity (AUC, four to 16-fold). In addition to the stronger activation, the maximal concentration was reached at a later time point (t_max_) and remained active for a longer period of time (t_1/2_). Only mRNA levels of IRF9 were predicted to decline faster in the cell culture experiment than under *in vivo* conditions.

### Interspecies variability in IFN-α drug action

In biomedical research, *in vitro* assays and animal models are often employed to estimate the interaction between human physiology and a drug [26]. This is due to the fact that many experiments in humans are obviously limited and invasive sampling in human tissue is hence challenging [27]. Most commonly, rats and mice are often used as a model organisms to represent the *in vivo* situation; however, this can lead to erroneous conclusions. Seok et al. [28] reported that, e.g., the immune response in mice and humans shows only poor correlation.

**Fig 8:**
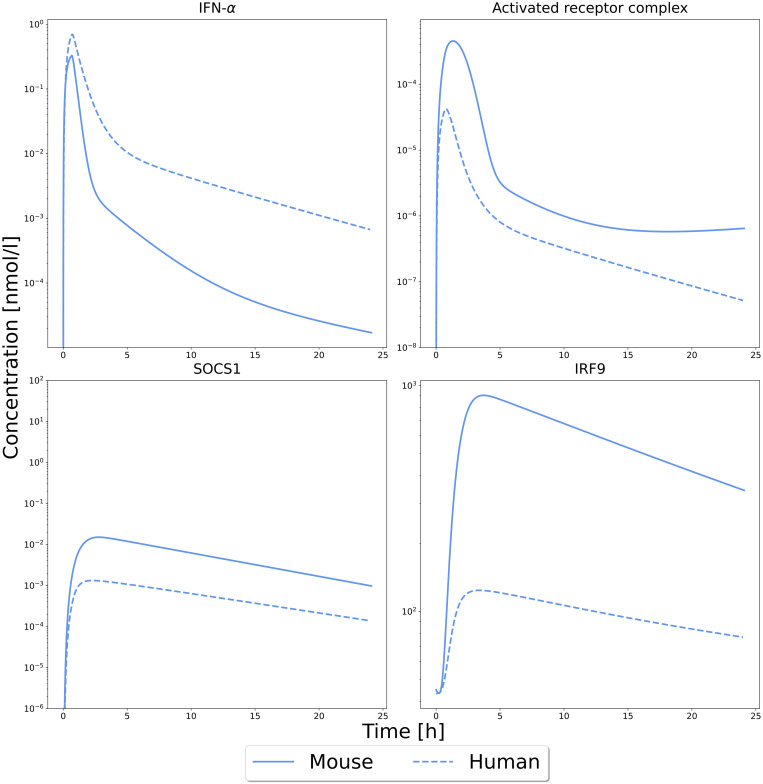
Predictions of a human and murine QSP for IFN-α. Predictions of human fit 5 (dashed line) from Kalra et al. [9] and the here established mouse model (solid line) for the same dose per body weight stimulation with IFN-α. Shown are the concentration of IFN-α in the interstitial space of the liver, the activation of the receptor complex, formation of the transcription factor ISGF3 and its binding to the target genes as well as transcription and translation of IRF9 and SOCS1.

**Fig 9:**
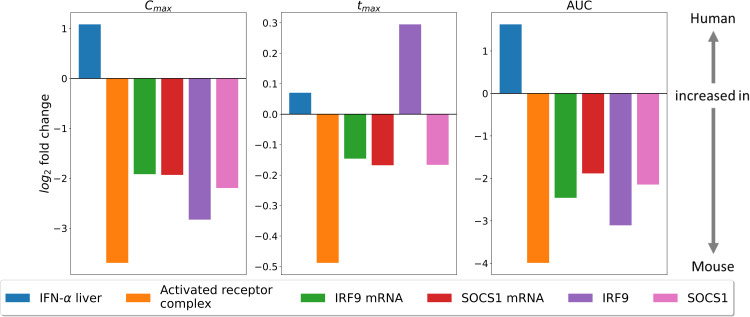
Fold change difference of predicted signalling patterns between a human and a mouse PBPK/PD. Fold change differences of signalling patterns predicted by the human ensemble from Kalra et al. 2019 [9] and the here established mouse model for the same dose per body weight stimulation with IFN-α. For evaluation of the differences the maximum concentration (C_max_, left), the time point when C_max_ is reached (t_max_, middle) and the area under the curve (AUC, right) were calculated for the concentration of IFN-α in the interstitial space of the liver, the activated receptor complex as well as SOCS1 and IRF9 mRNA and protein. Fold changes of these metrics were then calculated from all ten human models to the mouse model, and then averaged. Means are shown as log_2_ values, where positive fold changes indicate higher levels (C_max_, AUC) or later timing (t_max_) in human, and negative values the same in mice.

To compare the dynamic behavior of various key endpoints (IFN-α in the interstitial space of the liver, activated receptor complex, SOCS1, IRF9) we hence simulated both the published human and the new murine QSP model. In both cases, the same dose per body weight 3.0 µg/kg of murine and human IFN-α, respectively) was considered 40 minutes after i.v. injection. For simplicity, only one of the ten human predictions are shown (Fig 8), but all were considered in the assessment of cross-species differences of IFN-α signalling patterns between human and mice (Fig 9). The predicted dynamical behavior of the intracellular signalling pathway was overall comparable between human and mice. However, IFN-α levels in the interstitial space of the liver were much higher as well as more sustained in human than in mice (Fig 8). In contrast, downstream components of the signalling cascade showed an eight to 16-fold stronger response in the murine liver. In our analysis this is due to the efficient IFN- α binding resulting from the parameterizations after fitting (Table C in S2 Text). However, this could be also caused by a higher receptor abundancy in mice. With the current data, we are not able to separate these two parameters.

## Discussion

In this work, we established a new QSP mouse model in response to injected IFN-α doses. We used this model to systematically compare *in vitro* and *in vivo* drug responses of IFN-α injections in mice and compare these to humans. The model combines the upstream IFN-α pharmacokinetics and the downstream pharmacodynamics of the JAK/STAT pathway. As a functional readout, we used the antiviral response biomarker Mx2, which was measured both *in vitro* and *in vivo* in a mouse model. The PBPK part of the mouse model was carefully established and validated with PK data measured for different published doses of IFN-α [17,19] (low dose measured for this study) and IFN-β [18]. The latter was still used for establishing the PK model of IFN-α since there was a scarcity of data and the physicochemical properties of both IFN forms were very similar, including comparable molecular weights (~19–21 kDa), isoelectric points (pI ~ 5–7), and hydrodynamic radii, which support similar distribution and clearance mechanisms in vivo [29]. Thus, it was assumed that the PK profiles would be very similar as well. In addition, the intracellular part of the model was carefully validated with *in vitro* data on luciferase expression for IFN-α medium concentrations covering three orders of magnitude.

Subtype-specific PD effects (e.g., receptor binding dynamics, prolonged STAT1 activation) remain separable from PK and are not addressed in this work. Adding these details would primarily affect the pharmacodynamics (PD). For example, receptor affinity differences (e.g., IFN-β’s 1000-fold higher IFNAR1 affinity) influence PD, they have minimal impact on physicochemical-driven PK parameters like volume of distribution or renal clearance. Future work will incorporate subtype-specific receptor binding kinetics to refine PD predictions [30,31].

An obvious short-coming of the resulting models is the fact that only sparse data has been available for fitting and validation. As a result, kinetic parameters are not identifiable. To remedy this problem – at least to a certain extent – we employed model ensembles containing models with different parameterizations, all only constraint by the experimental data. With robust results from these model ensembles we can at least ensure that the findings are not an outcome of single arbitrarily chosen parameters. However, without doubt, all findings need additional experimental validations before an application in an applied setting is tried. This is all the more true, since so far – and to our knowledge – no data exists that directly compare, e.g., the molecular abundancies of signalling components between human and mouse models during interferon administration.

Of particular note, the resulting combined QSP model was able to predict *in vivo* measurements of Mx2 luciferase activity in the liver of mice with high accuracy. This is a strong indication for the overall model quality and provides confidence that the model can be used for further analysis. The newly developed mouse QSP model was first used to compare biomarker responses in cell culture assays with the *in vivo* situation. To this end, similar drug concentrations were considered in the cell culture medium and in the liver of the mouse PBPK model. It was found that pathway activity was stronger in the *in vitro* situation than under *in vivo* conditions. This can be attributed to the more sustained exposure level in cell cultures, where no physiological clearance occurs. This clearly emphasizes the need for modern organs-on-a-chip devices or similar to better reproduce the *in vivo* situation *in vitro*. The mouse QSP model was next compared to a previously established human QSP model [9] for interspecies comparisons. For identical weight-normalized doses, simulations showed higher IFN-α levels in the human liver. In contrast, the resulting responses in the downstream signalling cascade were found to be higher in mice. Thus, although the dynamics of the responses are comparable, clearly, the anti-viral response in mice is higher than in humans and would therefore be overestimated when using a mice model to extrapolate the human response. Mice are considered a useful model to investigate the antiviral immune response in humans, though inter-species differences have been identified from targeted gene deletions [32]. For example, STAT4 activation is necessary in humans for IFN-α induced development of type 1 T helper cells in response to viral infections. In contrast, IFN-α can neither activate STAT4 nor induce type 1 T helper cells in mice [32]. Viral susceptibility in mice and men also changes specifically along the JAK/STAT signaling pathway since STAT1 deficiency leads to a more severe phenotype in humans relative to mutations in IFNAR1/2, while deficiencies in STAT1 show a comparable viral phenotype to IFNAR1/2 deficiency in mice [33].

In pharmaceutical industry and academic research, scarcity of data is a common problem. Measurements for the efficacy of novel drug candidates in human are limited due to ethical and practical concerns. Instead, *in vitro* assays as well as animal models are often used as initial proof of concepts. However, there are also limitations in animal experiments as they are time-consuming, expensive, ethically concerning and their extrapolation to a clinically relevant context might be non-trivial. In this respect, QSP modelling is particularly valuable in understanding drug action of novel drug candidates. QSP models, as the one developed in this study, allow the continuous integration of such scarce and heterogeneous datasets, commonly generated along the drug development process, within a single computational model. There have been numerous preclinical and clinical studies to evaluate the clinical efficacy of IFN-α [34–37]. While it would be interesting to apply our models in the support of such analyses, it should be noted that our model describes only a part of the rather diverse IFN-α mediated immune response. Amongst others, ISGF3 is a transducer and activator of various interferon-stimulated response elements. These elements modulate in turn ISGs which, altogether, regulate a few hundred genes [38]. Expression characteristics of ISGs are an important signatures in several complex diseases such as specific rheumatic conditions as well as in different types of cancer [39]. This comes in addition to the role of ISGs in antiviral programmes during the host immune response [38]. For actual clinical applications of our QSP model further extensions and specifications are therefore necessary to support the *in silico* prediction of clinical trial outcomes - after significant further validation and data integration. The here established QSP models could then be used to analyze the variability in responsiveness to IFN-α therapy - at clinical, preclinical and cellular levels. Likewise, new approaches in immunooncology aim to boost the efficacy of immune checkpoint inhibitors or chimeric antigen receptor T cells by combination therapy with cytokines such as IFN-α, which also possesses antitumoral activity [40]. However, combination of two or more drugs increases the number of possible combinations and dose regiments to be tested in clinical trials.

QSP models can be used to to predict clinical drug effects and therefore reduce the number of patients required for study completion, thus enabling studies that would otherwise be limited by the availability of eligible patients [41]. We hence believe that QSP models will significantly support future drug development by supporting decision-making and study planning at critical steps.

In summary, we established a first QSP model for IFN-α administration in mice that includes the distribution of interferon in the body of the mice and the anticipated intracellular response in hepatocytes. Model parametrizations were based on model fitting to experimental data. Due to limited data being available, resulting in parameter non-identifiabilities, we used model ensembles with different parametrizations constraint by the experimental data to simulate the respective behavior. We compared the results with those achieved for a human QSP model and observed that less normalized IFN-α is expected to arrive at the liver in the mouse, nevertheless leading to a stronger intracellular response. The model will be easily extendable and adjustable to encompass further processes, answering different questions.For an applied setting of the model, further validations are needed, particularly to reduce some of the parameter uncertainties.

## Supporting information

S1 Text Cellular model. (DOCX)

S2 Text Pharmacokinetic model. (DOCX)

S3 Text QSP model. (DOCX)

S1 Data (Figs 3, 4 and 6). (XLSX)

## References

[1] Eddershaw P, Beresford A, Bayliss M. ADME/PK as part of a rational approach to drug discovery. Drug Discov Today. 2000;5(9):409–14. doi: 10.1016/s1359-6446(00)01540-3 10931658

[2] Meibohm B, Derendorf H. Pharmacokinetic/pharmacodynamic studies in drug product development. J Pharm Sci. 2002;91(1):18–31. doi: 10.1002/jps.1167 11782894

[3] Kuepfer L, Niederalt C, Wendl T, Schlender J-F, Willmann S, Lippert J, et al. Applied concepts in PBPK modeling: how to build a PBPK/PD model. CPT Pharmacometrics Syst Pharmacol. 2016;5(10):516–31. doi: 10.1002/psp4.12134 27653238 PMC5080648

[4] Niederalt C, Kuepfer L, Solodenko J, Eissing T, Siegmund H-U, Block M, et al. A generic whole body physiologically based pharmacokinetic model for therapeutic proteins in PK-Sim. J Pharmacokinet Pharmacodyn. 2018;45(2):235–57. doi: 10.1007/s10928-017-9559-4 29234936 PMC5845054

[5] Cook D, Brown D, Alexander R, March R, Morgan P, Satterthwaite G, et al. Lessons learned from the fate of AstraZeneca’s drug pipeline: a five-dimensional framework. Nat Rev Drug Discov. 2014;13(6):419–31. doi: 10.1038/nrd4309 24833294

[6] Danhof M. Systems pharmacology - Towards the modeling of network interactions. Eur J Pharm Sci. 2016;94:4–14. doi: 10.1016/j.ejps.2016.04.027 27131606

[7] Chen J, Li Y, Lai F, Wang Y, Sutter K, Dittmer U, et al. Functional comparison of interferon-α subtypes reveals potent Hepatitis B virus suppression by a concerted action of interferon-α and interferon-γ signaling. Hepatology. 2021;73(2):486–502. doi: 10.1002/hep.31282 32333814

[8] Hepgul N, Cattaneo A, Agarwal K, Baraldi S, Borsini A, Bufalino C, et al. Transcriptomics in interferon-α-treated patients identifies inflammation-, neuroplasticity- and oxidative stress-related signatures as predictors and correlates of depression. Neuropsychopharmacology. 2016;41(10):2502–11. doi: 10.1038/npp.2016.50 27067128 PMC4983179

[9] Kalra P, Brandl J, Gaub T, Niederalt C, Lippert J, Sahle S, et al. Quantitative systems pharmacology of interferon alpha administration: A multi-scale approach. PLoS One. 2019;14(2):e0209587. doi: 10.1371/journal.pone.0209587 30759154 PMC6374012

[10] Maiwald T, Schneider A, Busch H, Sahle S, Gretz N, Weiss TS, et al. Combining theoretical analysis and experimental data generation reveals IRF9 as a crucial factor for accelerating interferon α-induced early antiviral signalling. FEBS J. 2010;277(22):4741–54. doi: 10.1111/j.1742-4658.2010.07880.x 20964804

[11] Pulverer JE, Rand U, Lienenklaus S, Kugel D, Zietara N, Kochs G, et al. Temporal and spatial resolution of type I and III interferon responses in vivo. J Virol. 2010;84(17):8626–38. doi: 10.1128/JVI.00303-10 20573823 PMC2919002

[12] Lipps C, Klein F, Wahlicht T, Seiffert V, Butueva M, Zauers J, et al. Expansion of functional personalized cells with specific transgene combinations. Nat Commun. 2018;9(1):994. doi: 10.1038/s41467-018-03408-4 29520052 PMC5843645

[13] Seglen PO. Preparation of isolated rat liver cells. Methods Cell Biol. 1976;13:29–83. doi: 10.1016/s0091-679x(08)61797-5 177845

[14] Hoops S, Sahle S, Gauges R, Lee C, Pahle J, Simus N, et al. COPASI--a complex pathway simulator. Bioinformatics. 2006;22(24):3067–74. doi: 10.1093/bioinformatics/btl485 17032683

[15] Klipp E, Liebermeister W. Mathematical modeling of intracellular signaling pathways. BMC Neurosci. 2006;7(Suppl 1):S10. doi: 10.1186/1471-2202-7-S1-S10 17118154 PMC1775040

[16] Burnham KP. ADR. Model selection and multimodel inference. A practical information-theoretic approach. 2004. doi: 10.1007/978-0-387-22456-55

[17] Bohoslawec O, Trown PW, Wills RJ. Pharmacokinetics and tissue distribution of recombinant human alpha A, D, A/D(Bgl), and I interferons and mouse alpha-interferon in mice. J Interferon Res. 1986;6(3):207–13. doi: 10.1089/jir.1986.6.207 3745986

[18] Kiuchi Y, Yonehara M, Okada K, Suzuki J, Kobayashi S. The kinetics of interferon clearance in mice: comparison of mouse and human interferon. Jikken Dobutsu. 1984;33(1):85–9. doi: 10.1538/expanim1978.33.1_85 6468509

[19] Rosztóczy I. Study of the in vivo priming effect of interferon in mice. J Gen Virol. 1986;67( Pt 12):2731–7. doi: 10.1099/0022-1317-67-12-2731 3794665

[20] Samuel CE. Antiviral actions of interferons. Clin Microbiol Rev. 2001;14(4):778–809, table of contents. doi: 10.1128/CMR.14.4.778-809.2001 11585785 PMC89003

[21] Verhelst J, Hulpiau P, Saelens X. Mx proteins: antiviral gatekeepers that restrain the uninvited. Microbiol Mol Biol Rev. 2013;77(4):551–66. doi: 10.1128/MMBR.00024-13 24296571 PMC3973384

[22] Sadler AJ, Williams BRG. Interferon-inducible antiviral effectors. Nat Rev Immunol. 2008;8(7):559–68. doi: 10.1038/nri2314 18575461 PMC2522268

[23] Asano A, Jin HK, Watanabe T. Mouse Mx2 gene: organization, mRNA expression and the role of the interferon-response promoter in its regulation. Gene. 2003;306:105–13. doi: 10.1016/s0378-1119(03)00428-1 12657472

[24] Kuepfer L, Peter M, Sauer U, Stelling J. Ensemble modeling for analysis of cell signaling dynamics. Nat Biotechnol. 2007;25(9):1001–6. doi: 10.1038/nbt1330 17846631

[25] Niederalt C, Wendl T, Kuepfer L, Claassen K, Loosen R, Willmann S, et al. Development of a physiologically based computational kidney model to describe the renal excretion of hydrophilic agents in rats. Front Physiol. 2013;3:494. doi: 10.3389/fphys.2012.00494 23355822 PMC3553339

[26] Ioannidis JPA. Extrapolating from animals to humans. Sci Transl Med. 2012;4(151):151ps15. doi: 10.1126/scitranslmed.3004631 22972841

[27] Grizzle WE, Bell WC, Sexton KC. Issues in collecting, processing and storing human tissues and associated information to support biomedical research. Cancer Biomark. 2010;9(1–6):531–49. doi: 10.3233/CBM-2011-0183 22112494 PMC3445033

[28] Seok J, Warren HS, Cuenca AG, Mindrinos MN, Baker HV, Xu W, et al. Genomic responses in mouse models poorly mimic human inflammatory diseases. Proc Natl Acad Sci U S A. 2013;110(9):3507–12. doi: 10.1073/pnas.1222878110 23401516 PMC3587220

[29] Piehler J, Thomas C, Garcia KC, Schreiber G. Structural and dynamic determinants of type I interferon receptor assembly and their functional interpretation. Immunol Rev. 2012;250(1):317–34. doi: 10.1111/imr.12001 23046138 PMC3986811

[30] Abraham AK, Kagan L, Kumar S, Mager DE. Type I interferon receptor is a primary regulator of target-mediated drug disposition of interferon-beta in mice. J Pharmacol Exp Ther. 2010;334(1):327–32. doi: 10.1124/jpet.110.167650 20406858 PMC2912044

[31] Platanias LC, Uddin S, Domanski P, Colamonici OR. Differences in interferon alpha and beta signaling. Interferon beta selectively induces the interaction of the alpha and betaL subunits of the type I interferon receptor. J Biol Chem. 1996;271(39):23630–3. doi: 10.1074/jbc.271.39.23630 8798579

[32] Mestas J, Hughes CCW. Of mice and not men: differences between mouse and human immunology. J Immunol. 2004;172(5):2731–8. doi: 10.4049/jimmunol.172.5.2731 14978070

[33] Meyts I, Casanova J-L. Viral infections in humans and mice with genetic deficiencies of the type I IFN response pathway. Eur J Immunol. 2021;51(5):1039–61. doi: 10.1002/eji.202048793 33729549 PMC8900014

[34] Chen L, Borozan I, Feld J, Sun J, Tannis L-L, Coltescu C, et al. Hepatic gene expression discriminates responders and nonresponders in treatment of chronic hepatitis C viral infection. Gastroenterology. 2005;128(5):1437–44. doi: 10.1053/j.gastro.2005.01.059 15887125

[35] Hayashi K, Fukuda Y, Nakano I, Katano Y, Yokozaki S, Toyoda H, et al. Poor response to interferon treatment for chronic hepatitis C in human immunodeficiency virus-infected haemophiliacs. Haemophilia. 2000;6(6):677–81. doi: 10.1046/j.1365-2516.2000.00444.x 11122395

[36] Prosperini L, Capobianco M, Giannì C. Identifying responders and nonresponders to interferon therapy in multiple sclerosis. Degener Neurol Neuromuscul Dis. 2014;4:75–85. doi: 10.2147/DNND.S42734 32669902 PMC7337239

[37] Stránský J, Rýzlová M, Stríteský J, Nĕmecek V. Why is there a poor response to combined therapy with interferon alfa 2b and ribavirin in patients with chronic hepatitis C? Cas Lek Cesk. 2002;141(11):351–4. 12099059

[38] McNab F, Mayer-Barber K, Sher A, Wack A, O’Garra A. Type I interferons in infectious disease. Nat Rev Immunol. 2015;15(2):87–103. doi: 10.1038/nri3787 25614319 PMC7162685

[39] Borden EC. Interferons α and β in cancer: therapeutic opportunities from new insights. Nat Rev Drug Discov. 2019;18(3):219–34. doi: 10.1038/s41573-018-0011-2 30679806

[40] Berraondo P, Sanmamed MF, Ochoa MC, Etxeberria I, Aznar MA, Pérez-Gracia JL, et al. Cytokines in clinical cancer immunotherapy. Br J Cancer. 2019;120(1):6–15. doi: 10.1038/s41416-018-0328-y 30413827 PMC6325155

[41] Chelliah V, Lazarou G, Bhatnagar S, Gibbs JP, Nijsen M, Ray A, et al. Quantitative systems pharmacology approaches for immuno-oncology: adding virtual patients to the development paradigm. Clin Pharmacol Ther. 2021;109(3):605–18. doi: 10.1002/cpt.1987 32686076 PMC7983940

