# Supplementary material for: A comparative computational analysis of IFN-alpha pharmacokinetics and its induced cellular response in mice and humans
Source: PLoS Comput Biol. 2025 Sep 25;21(9):e1013509. doi: 10.1371/journal.pcbi.1013509 (PMC12500084; doi:10.1371/journal.pcbi.1013509)
Supplement: S1 Text — (DOCX) [file pcbi.1013509.s001.docx]

**A comparative computational analysis of IFN-alpha pharmacokinetics and its induced cellular response in mice and humans**

Priyata Kalra^1,4,$^, Bastian Kister^1,2,$^, Rebekka Fendt^1,2^, Mario Köster^3^, Julia Pulverer^3^, Sven Sahle^1^, Lars Kuepfer^2,&^, Ursula Kummer^1&^

^1^Department of Modelling of Biological Processes, COS/BioQuant, Heidelberg University, Im Neuenheimer Feld Heidelberg, Germany

^2^Institute for Systems medicine with Focus on Organ Interaction, University Hospital RWTH Aachen, Pauwelsstrasse Aachen, Germany.

^3^Model System for Infection and Immunity, Helmholtz Centre for Infection Research, Braunschweig, Germany.

^4^Now at Simulations Plus, Lancaster, California, United States of America.

$ Shared first authorship

& Shared senior authorship: lkuepfer@ukaachen.de;

ursula.kummer@bioquant.uni-heidelberg.de

**S1 Cellular model description**

The model is reduced compared to the model by Maiwald et al. [1]. It comprises known key components of the JAK/STAT pathway and consists of 21 species and 20 kinetic reactions. Most of the reactions are described using mass action kinetics. The feedback mechanism as well as transcription has been modelled with more complex reaction kinetics. The proteins located in the cytoplasm or nucleus have the suffix *c* or *n* respectively. In signalling pathways, the number of catalysts and substrate molecules are in the same order of magnitude, it has been reasoned that mass action kinetics are able to replicate the qualitative behaviour of the system [2].

In the following, we briefly describe the structure of the model using the names defined in the model. The corresponding table serves as overview of the reactions (Table 1) and the kinetic parameters (Table 2). Homologies between human and mice protein (Table 3) justified the transfer of parameters of the human model to the mouse model.

The model simulation time is 24 hours. The degradation of IFN-α was modelled (R1) to account for the reported half life of IFN-α in the range of 4 to 8 hours [3, 4]. The IFN-α - receptor binding was described step-wise. First, the free IFN-α IFN_atSite binds to IFNAR subunit 2 (IFNAR2) forming a ligand receptor complex (IFNA_R2_Complex) (R2) which then binds to IFNAR subunit 1 (IFNAR1) to form a trimeric complex (Activated Receptor Complex) (R3). The activated trimeric receptor-ligand complex associates with STAT2 in the cytoplasm (STAT2c) to activate the phosphorylated STAT2c (Rec2) (R5). Then, the union of cytoslic STAT1 with STAT2 was incorporated to form the hetero-dimer of STAT1-STAT2 (Rec 21) (R6). Consequently, binding to IRF9 (IRF9c) occurs to form ISGF3 complex in the cytoplasm (ISGF3c) (R7).

Some species are transported independently into the nucleus, namely, STAT1 (STAT1c/n) (R17), STAT2 (STAT2c/n) (R18), IRF9 (IRF9c/n) (R19) and ISGF3 (ISGF3c/n) (R20). The transcription complex (ISGF3n) then binds to the genes with ISRE sites (Open ISGF-3n binding sites to Occupied ISGF-3n binding sites) (R21) to transcribe the mRNA of IRF9 (mRNAc) (R10), SOCS1 (mRNAc_SOCS}) (R8) and Mx2 (mRNAc_Mx2) (R12). The liberation of the DNA-bound transcription factor (Occupied ISGF-3n binding sites) by nuclear phosphatase refurbishes the individual molecules and freed the ISRE sites (IRF9n, STAT1n, STAT2n and Open ISGF-3n binding sites) (R22).

The mRNAc of IRF9, SOCS1 and Mx2 are translated into the respective proteins (IRF9, SOCS1 and Mx2) which are further degraded (R14,R23, R15 & R16}). The mRNAc degradation for IRF9 mRNAc, SOCS1 mRNAc and Mx2 mRNAc was included (R11, R9 R13).

Lastly, also included was the SOCS1 protein binding to the heterotrimeric receptor complex resulting in the freed receptor subunits and thus the exertion of the negative feedback (R4). The details of the reactions and their kinetic rate laws are summarised in table A.

**Table A. Reaction List.** Collective reaction list with individual reactions for both mouse models: the *in vitro* hepatocyte and the *in vivo* PBPK/PD model. The column with R# and Cell Compartment depict the reactions that are present in the *in vitro* hepatocyte model. The columns with M# and multiscale compartment do the same for the *in vivo* PBPK/PD model. The column reactions describe the modelled pathway along with the column kinetic rate law which gives insight on the kinetics used to model each step.


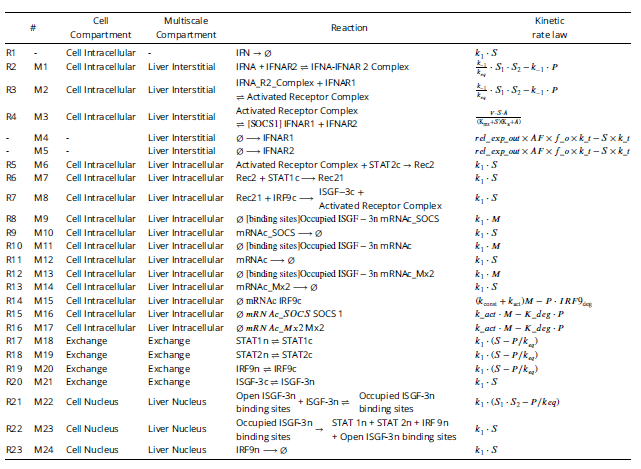


**Table B. Model parametrisations.** This table contains the parametrisation of the model ensemble describing IFN-α induced signalling through the JAK/STAT pathway.


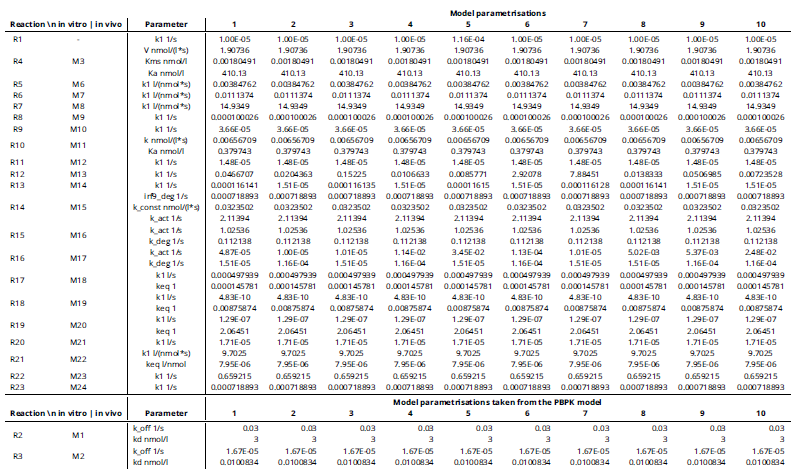


**Table C. BLASTP of human vs. mouse proteins of the JAK/STAT pathway.** This table recaps BLASTP (Query: Human, Subject explored: Mouse) for JAK/STAT signalling pathway proteins. The obtained sequence identities and homology are above 80 % for most proteins in the signalling pathway indicating a strong identity between the protein structures for the two species.


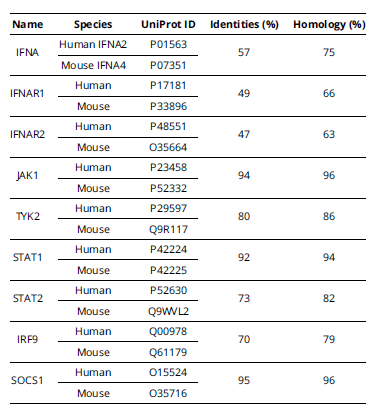


**Table D. Model parametrisations.** This table contains the parametrisation of a more diverse model ensemble describing IFN-α induced signalling through the JAK/STAT pathway.


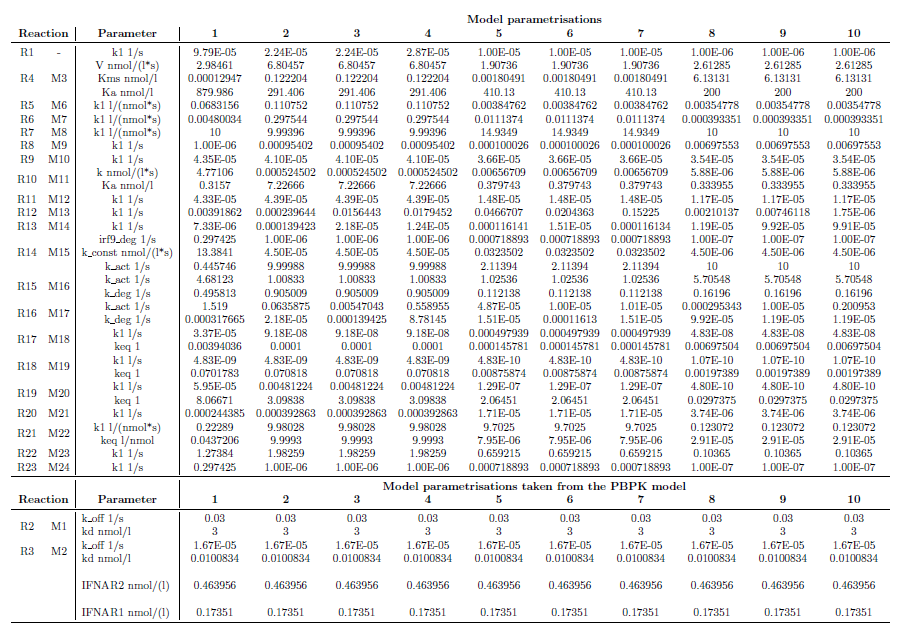


**Fig A.** Assessment of cooperative binding of the transcription factors and its effects on Mx2 expression following 5 U (top) and 500 U IFN-α (bottom) testing both Hill and mass action kinetics for parameter estimation.


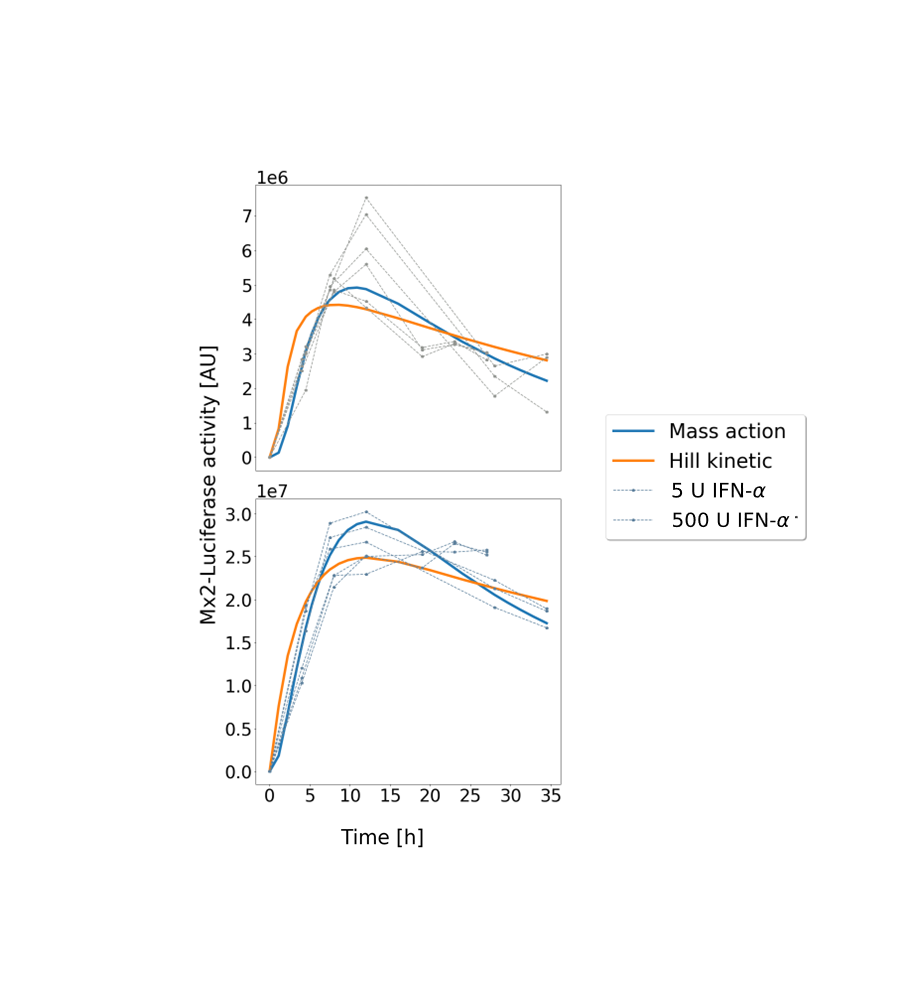


**Fig B.** Predictions of the whole model ensemble for Mx2 expression in response to 5 U or 500 U IFN-α simulation (solid lines) and corresponding data points used for fitting (dotted lines).


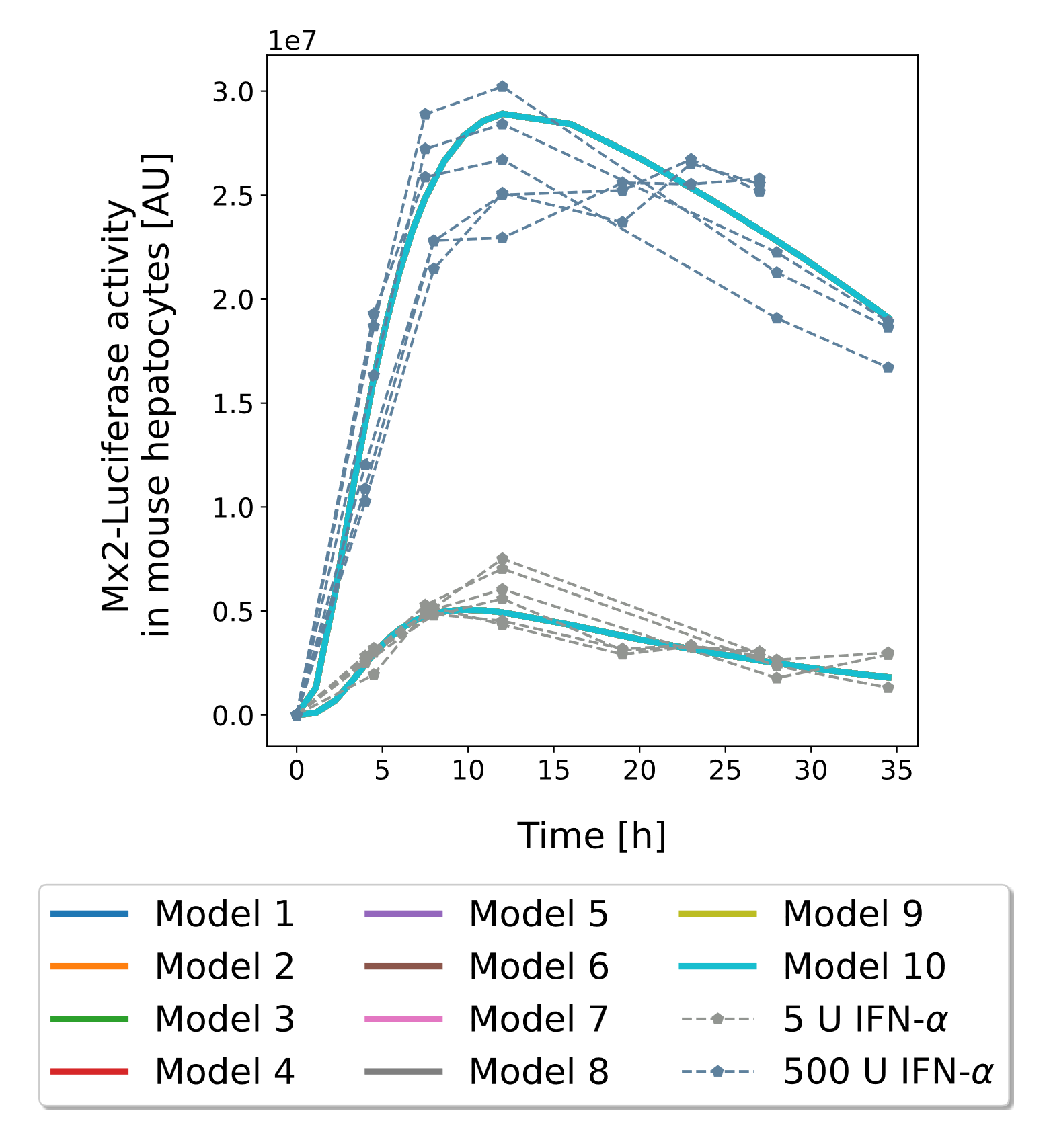


**Fig C.** Predicted dose responses of the model ensemble for Mx2 expression at 24 h after stimulation 0.13 nM, 0.26 nM, 1.3 nM, 2.6 nM, 6.5 nM, 13 nM, 26 nM and 65 nM IFN-α. (corresponding to 5 U, 10 U, 50 U, 100 U, 250 U, 500 U, 1000 U and 2500 U IFN-α).


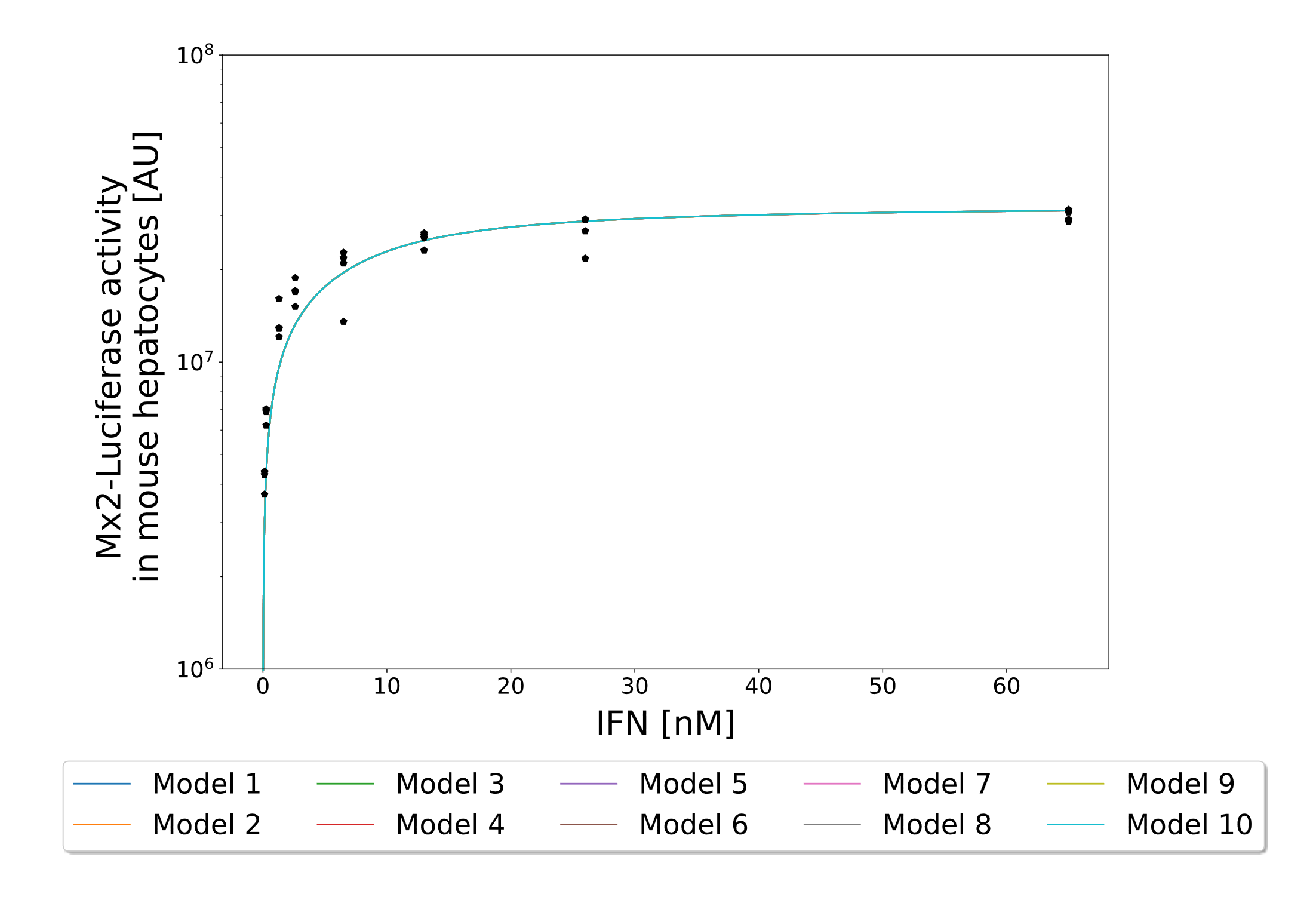


**Fig D.** Predictions of a more diverse model ensemble for Mx2 expression in response to 5 U or 500 U IFN-α simulation (solid lines) and corresponding data points used for fitting (dotted lines).


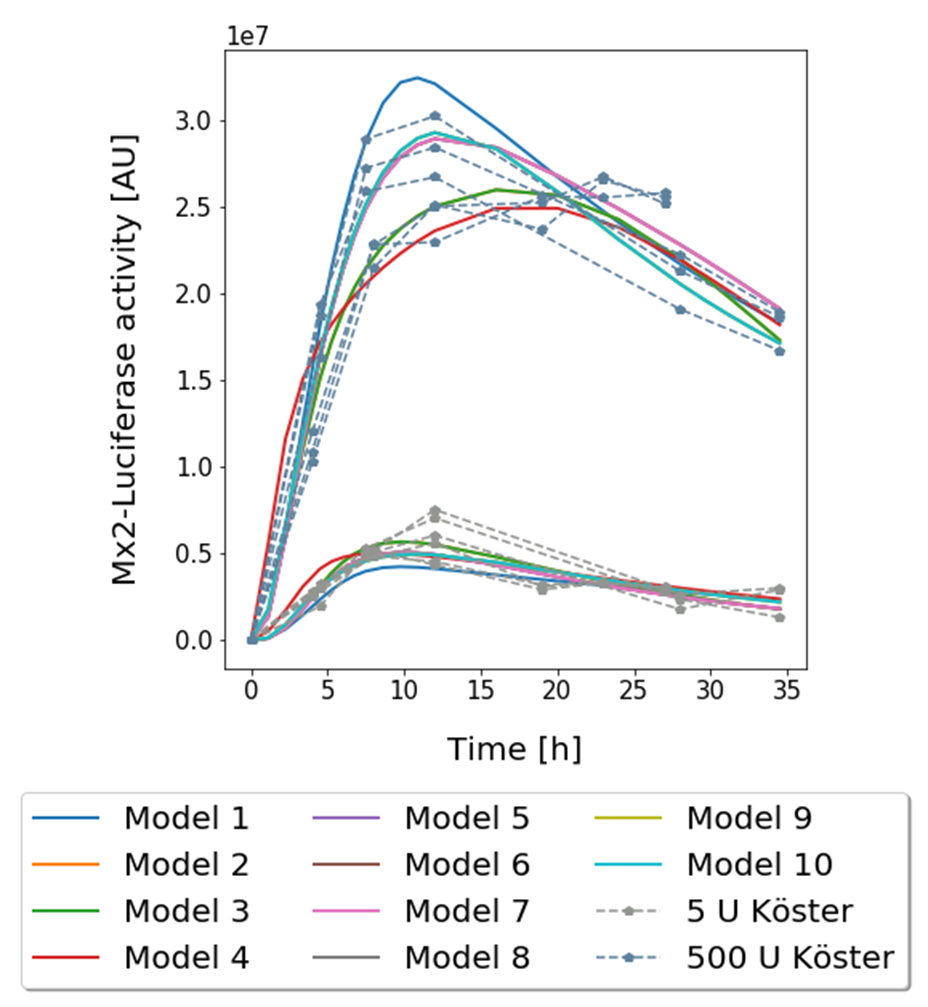


**Fig E.** Predicted dose responses of of a more diverse model ensemble for Mx2 expression at 24 h after stimulation 0.13 nM, 0.26 nM, 1.3 nM, 2.6 nM, 6.5 nM, 13 nM, 26 nM and 65 nM IFN-α. (corresponding to 5 U, 10 U, 50 U, 100 U, 250 U, 500 U, 1000 U and 2500 U IFN-α).


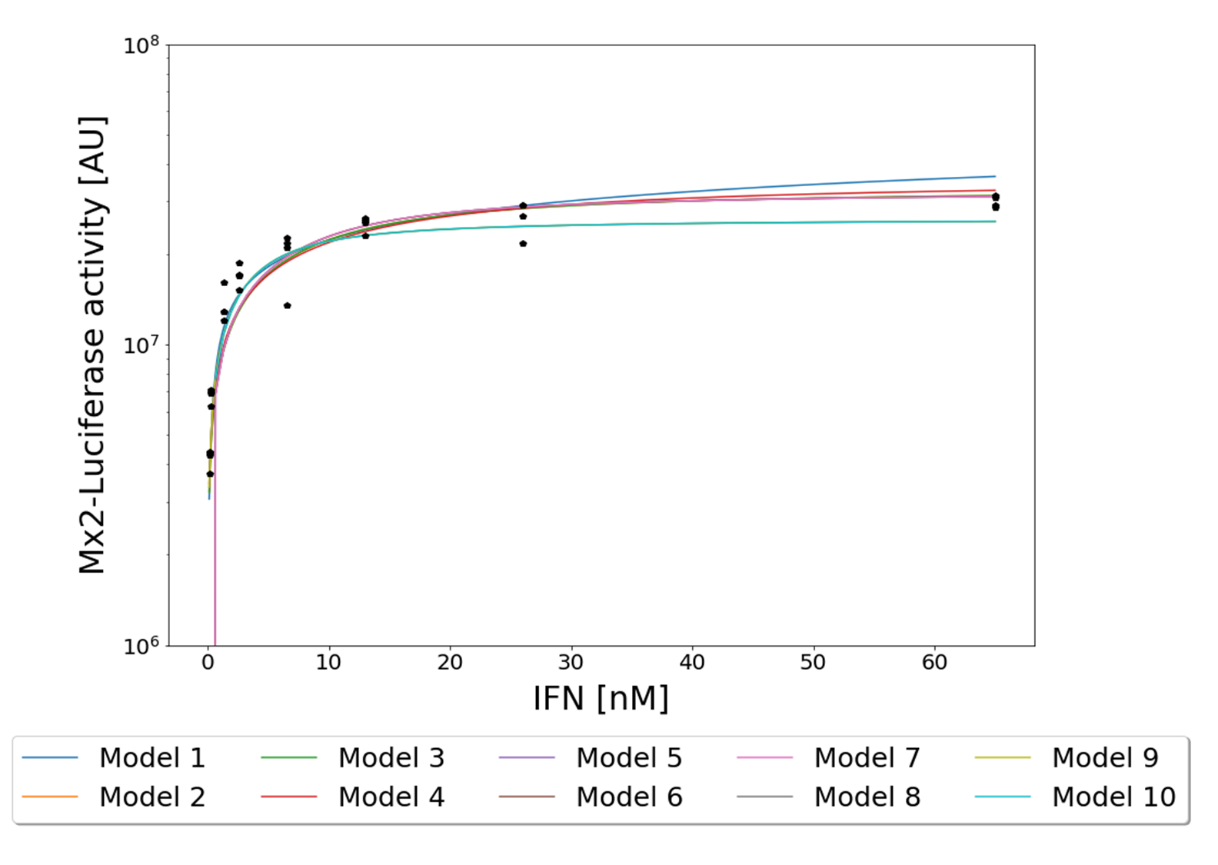


1. Maiwald T, Schneider A, Busch H, Sahle S, Gretz N, Weiss TS, et al. Combining theoretical analysis and experimental data generation reveals IRF9 as a crucial factor for accelerating interferon alpha-induced early antiviral signalling. FEBS J. 2010;277(22):4741-54. Epub 20101021. doi: 10.1111/j.1742-4658.2010.07880.x. PubMed PMID: 20964804.

2. Klipp E, Liebermeister W. Mathematical modeling of intracellular signaling pathways. BMC Neurosci. 2006;7 Suppl 1(Suppl 1):S10. Epub 20061030. doi: 10.1186/1471-2202-7-S1-S10. PubMed PMID: 17118154; PubMed Central PMCID: PMCPMC1775040.

3. Branca AA, Faltynek CR, D'Alessandro SB, Baglioni C. Interaction of interferon with cellular receptors. Internalization and degradation of cell-bound interferon. J Biol Chem. 1982;257(22):13291-6. PubMed PMID: 6292184.

4. Arnheiter H, Ohno M, Smith M, Gutte B, Zoon KC. Orientation of a human leukocyte interferon molecule on its cell surface receptor: carboxyl terminus remains accessible to a monoclonal antibody made against a synthetic interferon fragment. Proc Natl Acad Sci U S A. 1983;80(9):2539-43. doi: 10.1073/pnas.80.9.2539. PubMed PMID: 6302694; PubMed Central PMCID: PMCPMC393861.
