# Supplementary material for: A comparative computational analysis of IFN-alpha pharmacokinetics and its induced cellular response in mice and humans
Source: PLoS Comput Biol. 2025 Sep 25;21(9):e1013509. doi: 10.1371/journal.pcbi.1013509 (PMC12500084; doi:10.1371/journal.pcbi.1013509)
Supplement: S2 Text — (DOCX) [file pcbi.1013509.s002.docx]

**A comparative computational analysis of IFN-alpha pharmacokinetics and its induced cellular response in mice and humans**

Priyata Kalra^1,4,$^, Bastian Kister^1,2,$^, Rebekka Fendt^1,2^, Mario Köster^3^, Julia Pulverer^3^, Sven Sahle^1^, Lars Kuepfer^2,&^, Ursula Kummer^1&^

^1^Department of Modelling of Biological Processes, COS/BioQuant, Heidelberg University, Im Neuenheimer Feld Heidelberg, Germany

^2^Institute for Systems medicine with Focus on Organ Interaction, University Hospital RWTH Aachen, Pauwelsstrasse Aachen, Germany.

^3^Model System for Infection and Immunity, Helmholtz Centre for Infection Research, Braunschweig, Germany.

^4^Now at Simulations Plus, Lancaster, California, United States of America.

$ Shared first authorship

& Shared senior authorship: lkuepfer@ukaachen.de;

ursula.kummer@bioquant.uni-heidelberg.de

**S2 Pharmacokinetic** **model**

In this section we discuss the basic assumptions underlying the mathematical description of the pharmacokinetic model as well as the conversion of the literature date of IFN-α. The model parameters and the various literature sources used to describe the model are in Tables A and B in S1 Text.

**Calculation of literature IFN-α pharmacokinetic data**. To model the mouse pharmacokinetics, three plasma concentrations of murine IFN-α (mu-IFN-α ) were used in total, out of which two datasets were used for validation. The first was taken from Bohoslawec [1] where the mice were injected with a bolus intravenous injection of mu-IFN-α (subtype unknown). The dose was 8.7*10^5^ U. For each measurement time point, 3 mice were killed to yield the average of the data. mu-IFN-α concentrations were quantified by an antiviral assay and this concentration data was used in the parameter estimation of the PBPK model.

C57BL/6 Mx2-Luc transgenic mice were injected with an intravenous bolus dose of 5000U mu-IFN-α -4. Before sacrificing the mice were injected with luciferin so that the Mx2-Luciferace activity in the liver could be measured. They were then sacrificed and Mx2-Luciferase activity was measured by light emission from the organs of the mouse body. The residual plasma mu-IFN-α -4 in the serum was measured for 20 mins and the sample concentration was determined by ELISA. The specific activity of mu-IFN-α -4 was 1.4*10^8^ U/mg; the conversion of the IU to concentrations are described above. This dataset was used in preliminary analysis as it was for a short-time scale of 20 minutes.

The third dataset was digitized from Rosztoczy et al. [2] where an i.v. bolus dose of 2000 IU/g of mu-IFN-α was injected into male CFP mice with weight 20 ± 1 g. The specific activity of mu-IFN-α used in this publication was 1.2*10*7 U/mg. Data (an average of a set of six mice) was collected over a time period of 12 hours post injection.

For validation, the first dataset was extracted and digitized from the publication Kiuchi et al. [3]. In this work, a mix mu-IFN-α and mu-IFN-β was injected in male CDF1 mice as i.v. dose of 1*10^5^ IU/mouse. Plasma volume was estimated as one tenth of the body weight and serum volume as 50% of the plasma volume. The specific activity of the mu-IFN-α used was 3*10^7^ IU/mg. Each data-point was a measurement of pooled sera of three mice and the measurements were taken for 3 hours.

**Table A. Pharmacokinetic data of mu-IFN-α published by different groups**. Pharmacokinetic data of mu-IFN-α published by different groups. The data was used to fit and validate the pharmacokinetic model using PK-Sim.


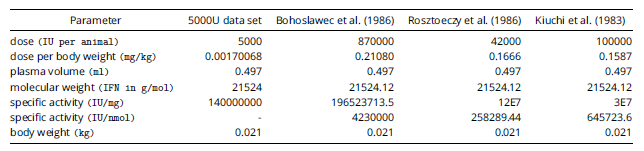


**PBPK reactions.** The clinical profile of IFN-α was modelled as zero order input rate and first order elimination rate. All the parameters for the pharmacokinetic modelling are listed in Table C. For modelling purposes, the liver is simplified as one big cell which is calculated on the size of the hepato-cellularity of the liver. The liver is divided into 3 compartments: the interstitial, the cytoplasm and the nucleus. The IFN receptor dynamics was modelled in the interstitial compartment and consists of detailed receptor ligand interactions along with receptor turnover. Remaining downstream reactions were compartmentalised as they were in the cellular signalling model (Table A in S1 Text).

**Calculation of receptor concentration.** A mean receptor density of approximately 0.55 molecules/µm^2^ has been measured, which corresponds to 500-1000 binding sites per cell [4-7]. The number of hepatocytes per gram of human liver has been estimated as 139*10^6^ cells/g liver [8]. It is known that the volume of the liver is 1.6 litres. With this information, total receptor concentration in the liver was calculated.


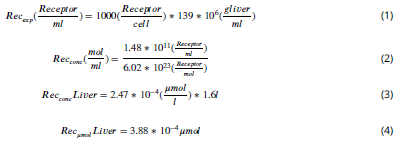


**Table B. IFN PK data used for PBPK model development**

| Bohoslawec et al. 1986 | |  | Rostoczy et al 1984 | |
| --- | --- | --- | --- | --- |
| 4.43 µg IFN-α | |  | 3.50 µg IFN-α | |
| Time[h] | conc [mM] |  | Time[h] | conc [mM] |
| 0.033 | 0.02743696 |  | 0.2 | 0.05089666 |
| 0.083 | 0.01642907 |  | 1.5 | 0.00025322 |
| 0.25 | 0.00972502 |  | 3 | 0.0001225 |
| 0.5 | 0.00496597 |  | 5.8 | 7.8754E-05 |
| 1 | 0.00041383 |  | 13.3 | 2.3635E-05 |
| 3 | 4.1383E-05 |  |  |  |

| Kuichi et al 1984 | |  |  |
| --- | --- | --- | --- |
| 3.33 µg IFN-α | |  |  |
| Time[h] | conc [mM] | conc [mM] | conc [mM] |
| 0.25 | 0.00368557 | 0.0013973 |  |
| 0.5 | 0.0011079 | 0.00088797 |  |
| 1 | 0.00040506 | 0.00031367 | 0.000667498 |
| 2 | 0.0001559 | 6.0193E-05 |  |
| 3 | 0.00016823 |  |  |

| own data |  |  |
| --- | --- | --- |
| 0.0357 µg mix of IFN-α and IFN-β | | |
| Time [h] | conc [mM] |  |
| 0.017 | 0.00015626 |  |
| 0.083 | 0.00011583 |  |
| 0.167 | 0.00010091 |  |
| 0.333 | 0.00010476 |  |

**Table C. Parameters of the mouse and human PBPK model.** This table contains the literature values and the fitted values of the pharmacokinetic parameters used in the mouse and human PBPK model.


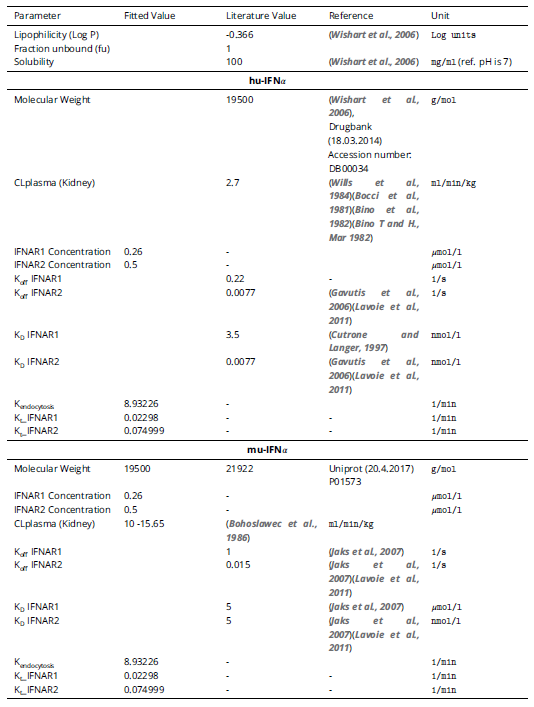


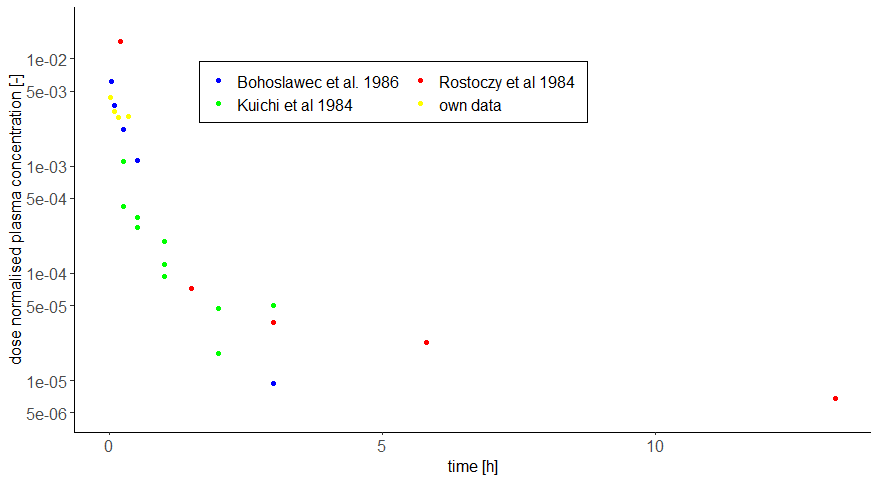


**Fig A. Dose normalized plasma concentration for the three IFN-α Pk profiles used in this study [1-3] and the additional own dataset generated for this study.** Dividing the observed time-concentration data with the administered dose (Table A) shows the general comparability of the four datasets.

1. Bohoslawec O, Trown PW, Wills RJ. Pharmacokinetics and tissue distribution of recombinant human alpha A, D, A/D(Bgl), and I interferons and mouse alpha-interferon in mice. J Interferon Res. 1986;6(3):207-13. doi: 10.1089/jir.1986.6.207. PubMed PMID: 3745986.

2. Rosztoczy I. Study of the in vivo priming effect of interferon in mice. J Gen Virol. 1986;67 ( Pt 12):2731-7. doi: 10.1099/0022-1317-67-12-2731. PubMed PMID: 3794665.

3. Kiuchi Y, Yonehara M, Okada K, Suzuki J, Kobayashi S. The kinetics of interferon clearance in mice: comparison of mouse and human interferon. Jikken Dobutsu. 1984;33(1):85-9. doi: 10.1538/expanim1978.33.1_85. PubMed PMID: 6468509.

4. Wilmes S, Beutel O, Li Z, Francois-Newton V, Richter CP, Janning D, et al. Receptor dimerization dynamics as a regulatory valve for plasticity of type I interferon signaling. J Cell Biol. 2015;209(4):579-93. doi: 10.1083/jcb.201412049. PubMed PMID: 26008745; PubMed Central PMCID: PMCPMC4442803.

5. Schreiber G, Piehler J. The molecular basis for functional plasticity in type I interferon signaling. Trends Immunol. 2015;36(3):139-49. Epub 20150214. doi: 10.1016/j.it.2015.01.002. PubMed PMID: 25687684.

6. Moraga I, Harari D, Schreiber G, Uze G, Pellegrini S. Receptor density is key to the alpha2/beta interferon differential activities. Mol Cell Biol. 2009;29(17):4778-87. Epub 20090629. doi: 10.1128/MCB.01808-08. PubMed PMID: 19564411; PubMed Central PMCID: PMCPMC2725717.

7. Francois-Newton V, Livingstone M, Payelle-Brogard B, Uze G, Pellegrini S. USP18 establishes the transcriptional and anti-proliferative interferon alpha/beta differential. Biochem J. 2012;446(3):509-16. doi: 10.1042/BJ20120541. PubMed PMID: 22731491.

8. Sohlenius-Sternbeck AK. Determination of the hepatocellularity number for human, dog, rabbit, rat and mouse livers from protein concentration measurements. Toxicol In Vitro. 2006;20(8):1582-6. Epub 20060629. doi: 10.1016/j.tiv.2006.06.003. PubMed PMID: 16930941.
