# Supplementary material for: A comparative computational analysis of IFN-alpha pharmacokinetics and its induced cellular response in mice and humans
Source: PLoS Comput Biol. 2025 Sep 25;21(9):e1013509. doi: 10.1371/journal.pcbi.1013509 (PMC12500084; doi:10.1371/journal.pcbi.1013509)
Supplement: S3 Text — (DOCX) [file pcbi.1013509.s003.docx]

**A comparative computational analysis of IFN-alpha pharmacokinetics and its induced cellular response in mice and humans**

Priyata Kalra^1,4,$^, Bastian Kister^1,2,$^, Rebekka Fendt^1,2^, Mario Köster^3^, Julia Pulverer^3^, Sven Sahle^1^, Lars Kuepfer^2,&^, Ursula Kummer^1&^

^1^Department of Modelling of Biological Processes, COS/BioQuant, Heidelberg University, Im Neuenheimer Feld Heidelberg, Germany

^2^Institute for Systems medicine with Focus on Organ Interaction, University Hospital RWTH Aachen, Pauwelsstrasse Aachen, Germany.

^3^Model System for Infection and Immunity, Helmholtz Centre for Infection Research, Braunschweig, Germany.

^4^Now at Simulations Plus, Lancaster, California, United States of America.

$ Shared first authorship

& Shared senior authorship: lkuepfer@ukaachen.de;

ursula.kummer@bioquant.uni-heidelberg.de

**S3 QSP model**


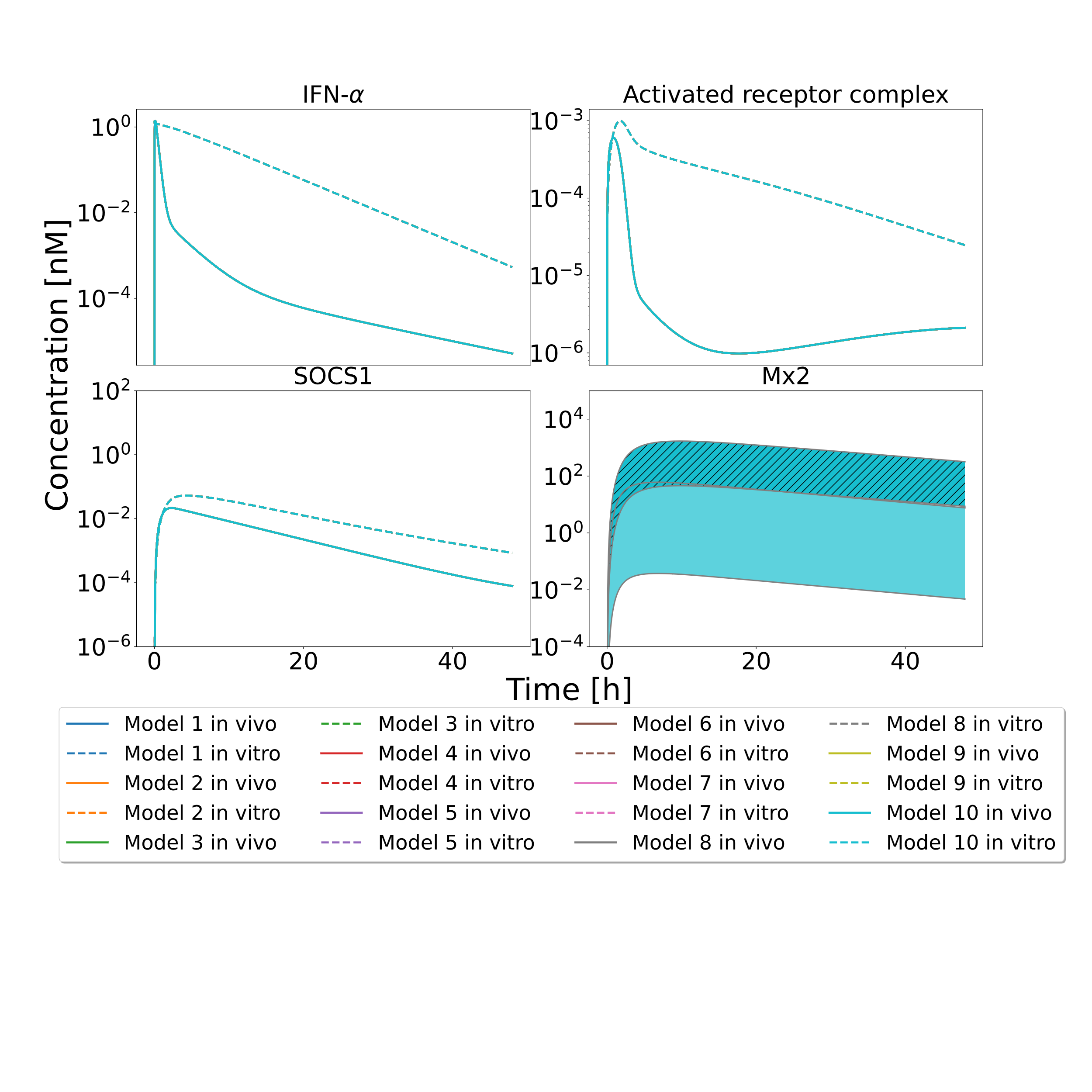
**Fig A. In vitro - in vivo difference in signalling patterns.** The difference in signalling pattern in the *in vivo* QSP response (1.43 µg IFN-α ; injected dose; solid lines) vs the *in vitro* PD response (0.695 model units; initial dose; dashed lines). Displayed are the time profiles of IFN-α in the interstitial space of the liver, the activated receptor complex, the negative regulator SOCS1 and the target protein Mx2. For Mx2 levels, the range of the ensemble's predictions are shown.
